# Requirement for A-type cyclin-dependent kinase and cyclins for the terminal division in the stomatal lineage of *Arabidopsis*

**DOI:** 10.1093/jxb/eru139

**Published:** 2014-03-31

**Authors:** Kezhen Yang, Hongzhe Wang, Shan Xue, Xiaoxiao Qu, Junjie Zou, Jie Le

**Affiliations:** Key Laboratory of Plant Molecular Physiology, Institute of Botany, Chinese Academy of Sciences, 20 Nanxincun, Beijing 100093, China

**Keywords:** *Arabidopsis*, cell cycle, stomatal development, symmetric division, transcription factor.

## Abstract

The *Arabidopsis* stoma is a specialized epidermal valve made up of a pair of guard cells around a pore whose aperture controls gas exchange between the shoot and atmosphere. Guard cells (GCs) are produced by a symmetric division of guard mother cells (GMCs). The R2R3-MYB transcription factor FOUR LIPS (FLP) and its paralogue MYB88 restrict the division of a GMC to one. Previously, the upstream regions of several core cell cycle genes were identified as the direct targets of FLP/MYB88, including the B-type cyclin-dependent kinase *CDKB1;1* and A2-type cyclin *CYCA2;3*. Here we show that *CDKA;1* is also an immediate direct target of FLP/MYB88 through the binding to *cis*-regulatory elements in the *CDKA;1* promoter region. CDKA;1 activity is required not only for normal GMC divisions but also for the excessive cell overproliferation in *flp myb88* mutant GMCs. The impaired defects of GMC division in *cdkb1;1 1;2* mutants could be partially rescued by a stage-specific expression of *CDKA;1*. Although targeted overexpression of *CDKA;1* does not affect stomatal development, ectopic expression of the D3-type cyclin *CYCD3;2* induces GC subdivision, resulting in a stoma with 3–4 GCs instead of the normal two. Co-overexpression of *CDKA;1* with *CYCD3*;2, but not with *CYCA2;3*, confers a synergistic effect with respect to GC subdivision. Thus, in addition to a role in stomatal formative asymmetric divisions at early developmental stages, CDKA;1 is needed in triggering GMC symmetric divisions at the late stage of stomatal development. However, timely down-regulation of CDKA;1–CYCD3 activity is required for restriction of GC proliferation.

## Summary

CDKA;1 is required for the termial divison of stomatal development. The results presented here use targeted expression to fine-tune the roles of CDKs and cyclins in regulating guard cell production.

## Introduction

In *Arabidopsis thaliana*, stomata consist of a pair of guard cells (GCs) surrounding a pore that permits gas exchange between internal plant tissues and the atmosphere. Stomata are produced through a series of divisions including at least one asymmetric and one symmetric division. The meristemoid mother cell (MMC) undergoes an asymmetric entry division to produce a small triangular-shaped meristemoid and a larger sister cell. Meristemoids usually undergo 1–3 rounds of asymmetric divisions and eventually differentiate into guard mother cells (GMCs). GMCs then divide once symmetrically to produce a pair of GCs (Bergmann and Sack, 2007). The final developmental stage of stomata is GC differentiation, pore formation, and GC shape control (Nadeau and Sack, 2002).

Three basic-helix–loop–helix (bHLH) transcription factors, SPEECHLESS (SPCH), MUTE, and FAMA, are required for the successive stages of stomatal development (Ohashi-Ito and Bergmann, 2006; MacAlister *et al.*, 2007; Pillitteri *et al.*, 2007). SPCH is essential for MMC formation, stomatal entry divisions, and maintenance of meristemoid stem cell activity (Pillitteri and Torii, 2012; Lau and Bergmann, 2012). MUTE promotes the transition of meristemoids into GMCs (Pillitteri *et al.*, 2007). Loss of function of *FAMA* causes tumours composed of stacked narrow cells, but overexpression of *FAMA* induces ectopic GCs without GMC divisions, suggesting the dual roles of FAMA in regulating differentiation and proliferation (Ohashi-Ito and Bergmann, 2006).

Two redundant R2R3 MYB transcription factors, FOUR LIPS (FLP) and MYB88, restrict the symmetrical cell division of the GMC (Lai *et al.*, 2005). Double mutants of *flp myb88* display stacked cells like *fama* mutants but some cells still could acquire the GC fate (Xie *et al.*, 2010). Reducing the activity of CYCLIN DEPENDENT KINASE B1;1 (CDKB1;1) by overexpression of the dominant-negative form *CDKB1;1.N161* or loss of function of both *CDKB1;1* and *CDKB1;2* (*cdkb1;1 1;2*) blocks the GMC symmetric division at the G_2_ to M phase transition of the cell cycle, resulting in the formation of single guard cells (SGCs) (Boudolf *et al.*, 2004*a*; Xie *et al.*, 2010). FLP/MYB88 can bind directly to a *cis*-regulatory element in the *CDKB1;1* promoter and can suppress *CDKB1;1* transcription levels. Although FAMA functions at the GMC to GC transition and regulates *CDKB1;1* expression as well, FAMA probably acts in a parallel pathway different from FLP/MYB88 (Ohashi-Ito and Bergmann, 2006).

Since CDK activation depends on its association with cyclin partners, co-expression of *CDKB1;1* and *CYCLIN A2;3* (*CYCA2;3*) enhances the kinase activity of CDKB1;1 and triggers ectopic cell divisions (Boudolf *et al.*, 2009). Defective GMC divisions are found in *cyca2* mutants, while the *cdkb1;1 cyca2;234* quadruple mutant displays more SGCs than *cyca2;234* triple mutants, though there is no phenotype in the *cdkb1;1* single mutant, suggesting that *CYCA2* and *CDKB1* genes synergistically promote GMC division. Consistently, a sustained high *CYCA2;3* and *CDKB1;1* expression is found in *flp myb88* epidermal tumours, indicating that FLP/MYB88 repress *CYCA2;3* and *CDKB1;1* transcription in a timely manner after GMC division to prevent cell overproliferation (Xie *et al.*, 2010; Vanneste *et al.*, 2011).

In yeast, there is only a single cyclin-dependent kinase, and its low, intermediate, or high activity is required for DNA replication licensing, DNA replication initiation, and cell mitosis, respectively (Porter, 2008). In *Arabidopsis*, the only one A-type CDK, CDKA;1 (also called CDC2A), which can rescue the fission yeast *cdc2* mutant, is required for cell cycle regulation during pollen, embryo, root, and shoot apical stem development (Inze and De Veylder, 2006; Gutierrez, 2008). Previous chromatin immunoprecipitation (ChIP)-chip experiments with antibodies against FLP/MYB88 revealed that the upstream promoter region of the *CDKA;1* gene might be the target for binding by FLP/MYB88 (Xie *et al.*, 2010), indicating a putative role for *CDKA;1* in stomatal GMC divisions.

In animals, CDK–cyclin complexes control G_1_ to S progression in the cell cycle through regulating phosphorylation of *RETINOBLASTOMA* proteins and releasing the E2F/DP complex (Harbour and Dean, 2000). Recently, the partial redundancy among the A- and B1-type CDKs in the *RETINOBLASTOMA-RELATED1* (*RBR1*; the homologue of the human tumour suppressor Retinoblastoma in *Arabidopsis*) regulatory network has been intensively investigated in plants. RBR1 is phosphorylated by several cyclin–CDK complexes, such as, CYCD6/CDKB1;1, CYCD3;1/CDKA;1, or CYCB1;1/CDKB1;1 (Cruz-Ramirez *et al.* 2012; Nowack *et al.*, 2012). However, ChIP experiments also revealed that *CDKB1;1* and *CDKB1;2* are the direct transcriptional targets of RBR1 (Nowack *et al.*, 2012). CYCD6 transcript levels are indirectly regulated by RBR1 through interaction with the SCARECROW (SCR) transcription factor, which in complex with SHORT ROOT regulates *CYCD6;1* expression during root stem cell asymmetric division (Cruz-Ramirez *et al.*, 2012). A negative regulatory feedback loop of RBR1 on CDK activity was also found in maize endosperm (Sabelli *et al.*, 2013).

Expression of *CDKA;1* in the stomatal lineage, under control of the *TOO MANY MOUTHS* (*TMM*) promoter, could partially rescue *cdkb1;1 1;2* stomata defects, suggesting functional redundancy between CDKA;1 and CDKB1s, in agreement with the arrested GMC divisions in the *cdka;1* null mutant (Weimer *et al.*, 2012). In addition, stomata in *RBR1* RNA interference (RNAi) lines often consist of four GCs (Borghi *et al.*, 2010). A relationship between two types of CDKs and RBR1 has been proposed for stomatal asymmetric division (Weimer *et al.*, 2012), but whether a similar network is required for the symmetric divisions during the last stage of stomatal development has not been well characterized.

It has been shown recently that FLP and MYB88 conditionally restrict the G_1_ to S transition during formation of stomata (Lee *et al.*, 2013). Here it is shown that *CDKA;1*, like *CDKB1;1*, is also a direct target of FLP/MYB88 through binding to the *cis*-regulatory elements in its promoter. CDKA;1 activity is required for both normal GMC division and cell overproliferation in *flp myb88* mutants.

## Materials and methods

### Plant materials and growth conditions

All genotypes were in a Columbia-0 (Col-0) ecotypic background. *Arabidopsis thaliana* plants were grown on soil or half-strength Murashige and Skoog (MS) medium at 22–24 °C with 16/8h light/dark cycles.

### Plasmid construction and plant transformation

The following constructs and transgenic plants were generated using the primers shown in Supplementary Table S1 available at *JXB* online: *pSPCH:CDKA;1*, *pSPCH:CYCD3;2*, *pMUTE:CDKA;1*, *pMUTE:CDKA;1.N146*, *pMUTE:CYCD3;2*, *pFAMA:CDKA;1*, *pFAMA:CDKA;1.N146*, *pFAMA:CYCD3;1*; *pFAMA:CYCD3;2*, *pFAMA:CYCD3;3*, and *pFAMA:CYCA2;3*. The promoter fragments of *SPCH*, *MUTE, FAMA*, as well as full-length cDNAs of *CDKA;1*, *CDKA;1.N146*, *CYCD3;1*, *CYCD3;2*, *CYCD3;3*, and *CYCA2;3* were obtained by PCR. All resulting DNA fragments were cloned into the pMD19-T vector (TaKaRa) and then subcloned into a pCAMBIA1300 vector (CAMBIA) and transformed into wild-type plants. Transgenic plants were selected on half-strength MS medium containing 25 μg l^–1^ hygromycin. Plants harbouring multiple constructs were created by crossing and confirmed by PCR. To obtain a viable *cdka;1* null mutant, a *pLAT52:CDKA;1* construct, in which the full-length cDNA of *CDKA;1* is driven by the pollen-specific *LAT52* promoter, was transformed into heterozygous *cdka;1* ^*+/–*^ plants (SALK_106809). The homozygosity of *cdka;1* plants was confirmed by PCR using the primers shown in Supplementary Table S1 at *JXB* online.

### *β*-Glucuronidase (GUS) staining

Before staining, young seedlings were incubated in 90% acetone for 2h at 4 °C. Seedlings were then washed in phosphate buffer and immersed in X-gluc solution (1mg ml^–1^ 5-bromo-4-chloro-3-indolyl β-d-glucuronide, 2mM ferricyanide, and 0.5mM ferrocyanide in 100mM phosphate buffer, pH 7.0) overnight at 37 °C in the dark. Seedlings were then cleared and imaged using an Olympus BX51 microscope.

### Cell viability assay

True leaves were immersed in a 0.1% neutral red solution for 15min at room temperature before imaging with an Olympus BX51 microscope.

### Microscopy

To obtain differential interference contrast (DIC) images, 2-week-old cotyledons were treated with destaining solution (containing 75% ethanol and 25% acetic acid) for 30min or overnight at room temperature until the chlorophyll was cleared. After a treatment with basic solution (7% NaOH in 60% ethanol) for 15min at room temperature, the samples were rehydrated via an ethanol series (40, 20, and 10%) for 15min at each step and then placed in 5% ethanol and 25% glycerol for 30min. Materials were mounted in 50% glycerol and imaged using an Olympus BX51 microscope. For fluorescence, samples were stained with 0.5% propidium iodide and fluorescence was imaged using a confocal laser scanning microscope (FV1000-MPE, Olympus).

### DAPI staining and measurement of DNA content

For analysis of nuclei in GCs, cotyledons were dissected and fixed in 70% ethanol for 3h, incubated in 4′,6-diamidino-2-phenylindole (DAPI) staining solution for at least 30min, and excited by UV fluorescence. To score relative DNA levels, the total integrated density of DAPI fluorescence from selected nuclei was analysed using ImageJ (http://rsb.info.nih.gov/ij/, last accessed 20 March 2014) with fluorescence from nearby areas subtracted to standardize relative fluorescence levels. GCs resembling those in the wild type as well as those in epidermal cells that were newly divided were used as the references.

### Real-time qPCR

Total RNA from 10-day-old seedlings were extracted using TRNzol reagent (http://www.tiangen.com, last accessed 20 March 2014). Reverse transcription was performed using a Promega kit (http://www.promega.com, last accessed 20 March 2014). Amplification of the *KAT1* gene was used as an internal control. Real-time quantitative PCR (RT-qPCR) experiments were repeated three times independently. The cDNA was amplified using SYBR Premix Ex Taq™ (TaKaRa) with a Corbett RG3000.

### Yeast one-hybrid assay

The region between base pairs 859 and 568 upstream of the *CDKA;1* gene was split into a 191bp upstream fragment and a 100bp downstream fragment, subjected to PCR, and ligated into the pLacZi2μ vector. *FLP* and *MYB88* cDNA were cloned into pB42AD. Yeast one-hybrid assays were performed as previously described (Tang *et al.*, 2012).

### Protein expression

Full-length cDNA sequences of *FLP* and *MYB88* were amplified by using the primers shown in Supplementary Table S1 at *JXB* online. *FLP* and *MYB88* cDNA fragments were then ligated into pET-28a. Fusion proteins were expressed in the BL21 (DE3) strain of *Escherichia coli* by induction with 0.1mM isopropyl-β-d-thiogalactopyranoside (IPTG) at 18 **°**C for 24h. His-FLP and His-MYB88 proteins were purified by Ni-NTA agarose (GE Healthcare) following the manufacturer’s instructions.

### Electrophoretic mobility shift assay (EMSA)

Oligonucleotide probes were synthesized and labelled with biotin at the 3′ end (Thermo Scientific) and the probe sequences are shown in Supplementary Table S1 at *JXB* online. EMSA was performed using a Light Shift Chemiluminescent EMSA kit (Thermo Scientific). Briefly, biotin-labelled probes were incubated in 1× binding buffer, 2.5% glycerol, 50mM KCl, 5mM MgCl_2_, 0.05% NP-40, and 10mM EDTA with or without proteins at room temperature for 20min. For probe competition controls, non-labelled probes were added to the reactions.

### Yeast two-hybrid assay

Gal4 system vectors were used for yeast two-hybrid assays (Clontech). The full-length coding sequences of *CDKA;1.N146* and *CYCD3;2* were amplified using the primers shown in Supplementary Table S1 at *JXB* online. cDNA fragments from *CYCD3;2* were cloned into a pGBKT7 vector, and then *CDKA;1.N146* cDNA fragments were cloned into a pGADT7 vector. Constructs were then co-transformed into an AH109 yeast strain and selected on SD/–Leu–Trp or SD/–Leu–Trp–His–Ade plates. X-Gal activity was analysed according to the manufacturer’s instructions (Clontech).

### Bimolecular fluorescence complementation (BiFC) assay

Coding sequences of *CDKA;1.N146* and *CYCD3;2* were amplified using the primers shown in Supplementary Table S1 at *JXB* online. Fragments were cloned into the pSPYNE-35S and pSPYCE-35S vectors (Walter *et al.*, 2004) to obtain *CYCD3;2-YN* and *CDKA;1.N146-YC*. *CYCD3;2-YN* and *CDKA;1.N146-YC* were co-transformed into *Arabidopsis* protoplasts and imaged using a laser scanning confocal microscope (FV1000-MPE, Olympus).

### Pull-down assays

Full coding sequences of *CDKA;1.N146* and *CYCD3;2* were amplified by using the primers shown in Supplementary Table S1 at *JXB* online. The sequences were then ligated into pET-28a and pGEX4T-1 vectors, respectively. Fusion proteins were expressed in the BL21 (DE3) strain of *E. coli* by induction with 0.2mM IPTG at 17 °C for 4h. The harvested culture (5000rpm, 15min, 4 °C) was re-suspended with ice-cold phosphate-buffered saline (PBS) and then lysed by sonication. The cell lysate was cooled to 4 °C and centrifuged at 10 000rpm for 15min. The glutathione *S*-transferase (GST)–CYCD3;2 supernatant was loaded onto 0.5ml of glutathione–Sepharose (GE Healthcare) and washed with PBS. The GST–CYCD3;2 fusion protein on glutathione–Sepharose (GE Healthcare) was then incubated with the His6-CDKA.N146 supernatant. After 1h incubation at 4 °C, the agarose was washed twice with PBS and eluted with 10mM reduced glutathione elution buffer. Elution samples were separated by 12% SDS–PAGE, transferred to a PVDF membrane (Millipore) using a semi-dry blotting system (Bio-Rad), and then incubated with an anti-His6 monoclonal antibody followed by AP-conjugated anti-mouse antibody. The colour reaction was performed using an NBT/BCIP Kit (Invitrogen).

## Results

### CDKA;1 activity is required for the late stages of stomatal development

**Fig. 1.**
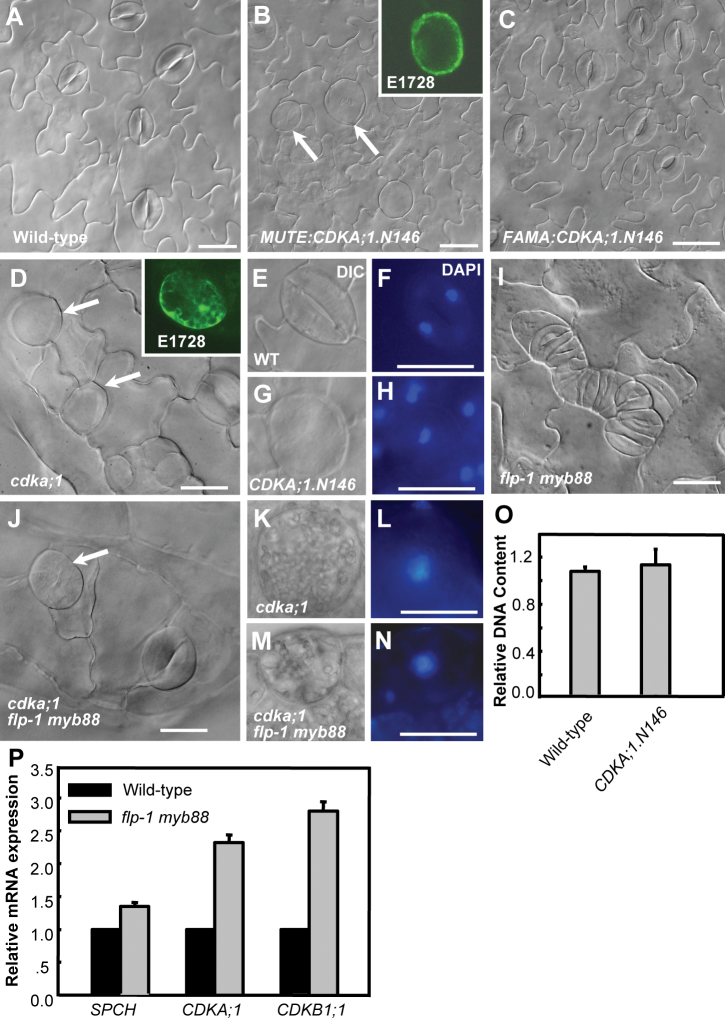
*CDKA;1* is required for excessive GMC divisions in *flp-1 myb88* mutants. (A) Wild-type epidermis. (B, C) The expression of a dominant-negative *CDKA;1.N146* construct driven by the *MUTE* promoter (*MUTE:CDKA;1.N146*), but not the *FAMA* promoter (*FAMA:CDKA;1.N146*), leads to formation of SGCs. Inset: expression of the mature GC marker E1728 in an SGC. (D) SGCs were found in loss-of-function *cdka;1* leaves. Inset: E1728. (E–H) Paired DAPI fluorescence micrographs and DIC images showing normal GCs in wild-type (E, F) and uninucleate SGCs in *MUTE:CDKA;1.N146* (G, H). (I) Stomatal clusters in the *flp-1 myb88* double mutant. (J) The *flp-1 myb88* cluster is repressed in the *cdka;1* background. An arrow indicates an SGC. (K–N) DAPI staining of SGCs in *cdka;1* and the *flp-1myb88 cdka;1* triple mutant. (O) Quantitative analysis of DAPI fluorescence revealed that *MUTE:CDKA;1.N146* SGCs contain a DNA content comparable with that of normal GCs. (P) Transcript levels of *SPCH*, *CDKA;1*, and *CDKB1;1* in *flp-1 myb88* double mutants. Bars=20 μm. (This figure is available in colour at *JXB* online.)

Stomata formation is greatly inhibited in *cdka;1* null mutant cotyledons, which demonstrates the essential role of *CDKA;1* in stomatal formative divisions. The occasional occurrence of arrested GMCs and expression of *CDKA;1* at late stages of the stomatal cell lineage suggests an involvement of CDKA;1 in the subsequent events as well (Xie *et al.*, 2010; Weimer *et al.*, 2012). Overexpression of a dominant-negative allele of *CDKA;1* (*CDKA;1.N146*) was previously used to reveal the function of *CDK* genes in plant development (Hemerly *et al.*, 1995; De Veylder *et al.*, 2000; Joubès *et al.*, 2004; Gaamouche *et al.*, 2010). *MUTE* and *FAMA* are specifically expressed at the transition phases of meristemoid to GMC and GMC to GC, respectively. Thus, the *CDKA;1.N146* construct was driven by the upstream regulatory regions of *MUTE* and *FAMA* genes. Targeted expression of either *MUTE:CDKA;1.N146* or *FAMA:CDKA;1.N146* did not affect the initiation of stomatal stem cells and morphogenesis of pavement cells (Fig. 1A–C). However, *MUTE:CDKA;1.N146* transgenic lines exhibit large abnormally shaped cells expressing a GC fate marker, E1728 (Gardner *et al.*, 2009), demonstrating that those round cells have acquired GC identity (inset in Fig. 1B).

Loss of *CDKA;1* function impairs the last mitotic division in the male gametophyte, leading to 50% of pollen with two gametes, and no homozygous *cdka;1* seedlings are produced in self-pollinated *cdka;1* ^*+/–*^ plants (Iwakawa *et al.*, 2006). Therefore, viable homozygous *cdka;1* seedlings were recovered from *cdka;1* ^*+/–*^ plants by transformation with *pLAT52:CDKA;1* constructs. As in *MUTE:CDKA;1.N146* seedlings, abnormal undivided round cells were also found in the leaves of homozygous *cdka;1* seedlings. Consistently, expression of the E1728 marker suggests that the undivided *cdka;1* GMCs could eventually also acquire GC cell fate (Fig. 1D).

Similar fluorescent intensity of DAPI-stained nuclei in normal 2-GC stomata and in nuclei of *MUTE:CDKA;1.N146* SGCs indicated that CDKA;1.N146 prevented DNA synthesis and caused cell cycle arrest (Fig. 1E–H, O), this is in contrast to the a 4C DNA content in SGCs from *cdkb1;1 1;2*, *cyca2;234* mutants or *35S:CDKB1;1.N161* transgenic plants (Xie *et al.*, 2010; Vanneste *et al.*, 2011). Little effect on the symmetric division was observed when the dominant-negative *CDKA;1* was activated later with the *FAMA:CDKA;1.N146* construct (Fig. 1C). Therefore, CDKA;1 activity is required for the early events, such as the G_1_ to S transition, in the GMC cell cycle.

### Identification of FLP/MYB88-binding sites in the *CDKA;1* promoter

The weak *flp-1* allele displays a stomatal cluster phenotype that often contains four stacked GCs caused by an extra round of division in a GMC (Lai *et al.*, 2005). The *flp-1 myb88* double mutant displays an enhanced stomatal phenotype with more and larger stomatal clusters (Fig. 1I). To determine whether CDKA;1 is required for the additional GMC divisions in the *flp-1 myb88* mutant background, *cdka;1 flp-1 myb88* triple mutants were generated. The typical GC stacks of the *flp-1 myb88* mutants were not found in triple mutants, instead SGCs are often present, suggesting that the cell overproliferation caused by mutations of *FLP* and *MYB88* required CDKA;1 activity (Fig. 1J). DAPI staining reveals a single nucleus in the round cells from the triple mutant, implying that arrested division is not due to a defective cytokinesis upon mitosis (Fig. 1K–N). Using the transcript level of a GC-specific gene *KAT1* as the internal reference for RT-qPCR analysis, it was found that the transcription of *CDKA;1*, like that of *CDKB1;1*, was up-regulated in *flp-1 myb88* plants, indicating that *FLP/MYB88* negatively regulate *CDKA;1* transcription (Fig. 1P).

**Fig. 2.**
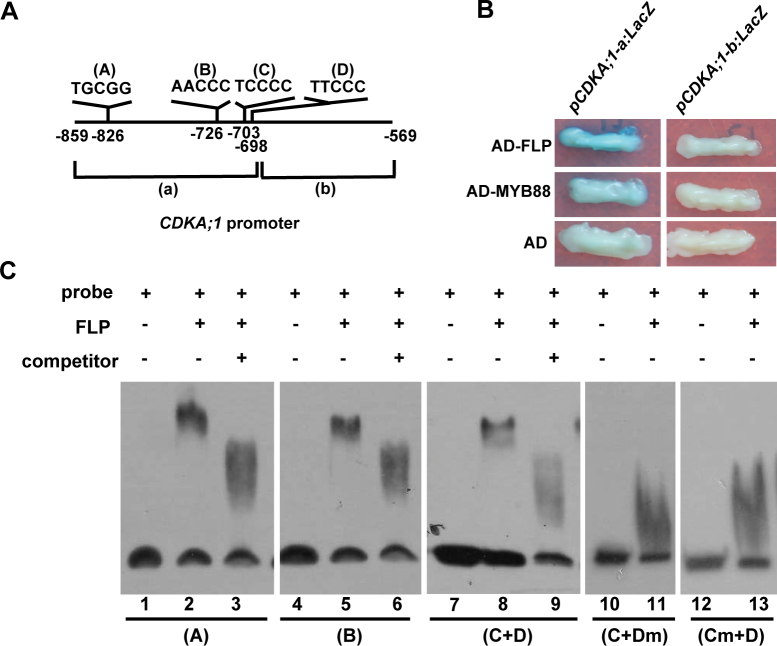
FLP directly binds the *CDKA;1* promoter. (A) Schematic diagram of four putative FLP/MYB88 *cis*-regulatory binding elements in the *CDKA;1* promoter, labelled as elements A–D. (B) Yeast-one-hybrid result shows that FLP and MYB88 can bind to fragment ‘a’ of the *CDKA;1* promoter. (C) EMSA results show that FLP can bind each binding element in *CDKA;1*. Lanes 1, 4, 7, 10, 12, probes only; lanes 2, 5, 8, 11, 13, probes with His-FLP fusion proteins; lanes 3, 6, 9, probes, His-FLP proteins, plus non-labelled competitors. Lanes 1–3, element A; lanes 4–7, element B; lanes 7–9, element C+D; lanes 10–13, nucleotides in element C or D were substituted by nucleotide A. (This figure is available in colour at *JXB* online.)

Previous ChIP-chip experiments with FLP/MYB88 antisera also revealed that the region between base pairs 859 and 568 upstream of the translational start site of the *CDKA;1* gene might be the target of FLP/MYB88 (Xie *et al.*, 2010). To identify the FLP/MYB88 direct binding sites in the *CDKA;1* promoter, the above promoter region was split into 191bp and 100bp fragments, named fragment ‘a’ and ‘b’, respectively (Fig. 2A). Yeast one-hybrid assay indicates that the binding sites are present within the fragment ‘a’ (Fig. 2B). Then the sequence in fragment ‘a’ was scanned and four putative FLP/MYB88 *cis*-regulatory binding elements were found (Xie *et al.*, 2010), namely TGCGG, AACCC, TCCCC, and TTCCC, termed elements A–D, respectively (Fig. 2A). Each of these sites was tested in EMSAs using tagged His-FLP or His-MYB88 fusion proteins. Since elements C and D are closely located within the *CDKA;1* promoter, all nucleotides within either the C or D elements were replaced with adenine in EMSA analysis. The EMSA results show FLP and MYB88 can interact with elements A, and B, and with a sequence composed of both the C and D elements (Fig. 2C; Supplementary Fig. S1 at *JXB* online).

### *CDKA;1* could rescue defective GMC divisions in *cdkb1* mutants

*CDKB1;1* and *FLP* show a similar expression pattern from the late GMC to the newly formed GC, in agreement with the role of FLP in regulating *CDKB1;1* transcription (Xie *et al.*, 2010). Targeted expression of *CDKB1;1* under the control of the *FAMA* promoter (*FAMA:CDKB1;1*) is sufficient to complement the SGC phenotype found in *cdkb1;1 1;2* double mutants (Table 1). The *CDKA;1* construct driven by *FAMA* was introduced into *cdkb1;1 1;2* double mutants. Interestingly, expression of this *FAMA:CDKA;1* could reduce the number of SGCs formed in *cdkb1;1 1;2*, indicating that *CDKA;1* can partially substitute for *CDKB1* genes in promoting GMC divisions (Table 1).

**Table 1.** Quantification of formation of single guard cells in cotyledon epidermis

Genotype	SGC (%)	SGC	Counts
Col	0	0	680
*cdkb1;1 1;2*	31	384	1238
*FAMA:CDKB1;1 cdkb1;1 1;2* line 1	0	0	964
*FAMA:CDKB1;1 cdkb1;1 1;2* line 2	0	0	1000
*FAMA:CDKA;1 cdkb1;1 1;2* line 1	22.5	231	1026
*FAMA:CDKA;1 cdkb1;1 1;2* line 2	17.6	115	650
*35S:CDKB1;1.N161*	13	169	1308
*FAMA:CYCA2;3 35S:CDKB1.N161*	2.3	35	1502
*FAMA:CYCD3;2 35S:CDKB1.N161*	0	0	852

### A- and D-type cyclins are rate limiting for division after the terminal stomatal division

**Fig. 3.**
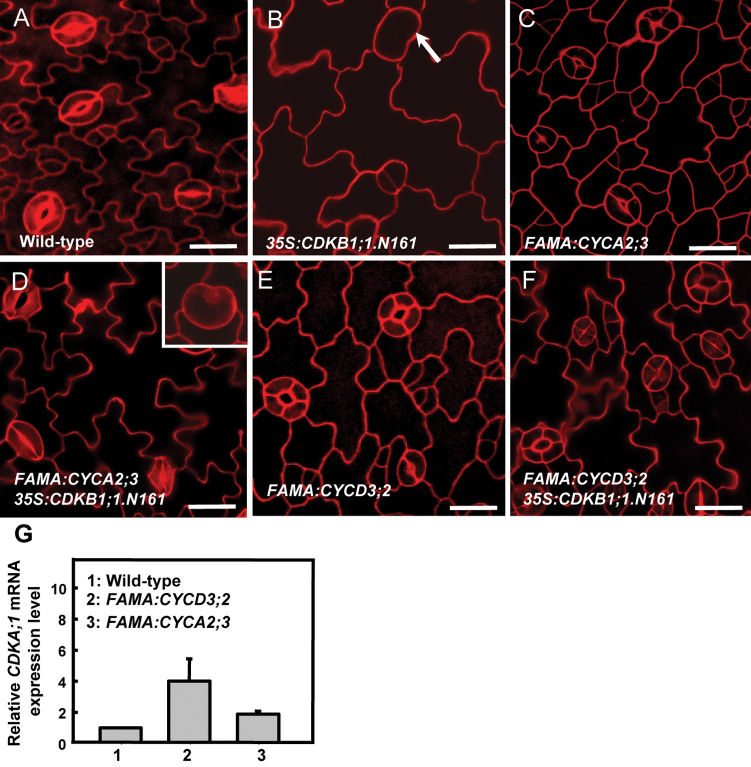
Overexpression of *CYCA2;3* and *CYCD3;2* can reduce the number of SGCs in *35S:CDKB1;1.N161*. (A) Wild-type epidermis. (B) SGCs were found in *35S:CDKB1;1.N161* epidermis. (C) *FAMA*:*CYCA2;3*-induced GC subdivisions, leading to stomata containing 3–4 GCs. (D) *FAMA:CYCA2;3* partially compensates the defects of GMC division in *35S:CDKB1;1.N161.* Inset: an occasionally found SGC (refer to Supplementary Fig. S4 at *JXB* online). (E) *FAMA:CYCD3;2* induces GCs to subdivide, leading to GCs with 3–4 stomata. (F) No SGC was found in *35S:CDKB1;1.N161* plants harbouring *FAMA:CYCD3;2.* (G)The *CDKA;1* transcript levels in *FAMA:CYCD3;2* and *FAMA:CYCA2;3* transgenic plants. Bars=20 μm. (This figure is available in colour at *JXB* online.)

Previous studies revealed that CDKB1;1 and CYCA2;3 form a functional complex regulating the mitosis to endocycle transition (Boudolf *et al.*, 2009). CYCA2s and CDKB1;1 contribute synergistically to the CDK activity required for GMC divisions (Vanneste *et al.*, 2011). The dominant-negative *CDKB1;1.N161* competes with the endogenous CDKs, leading to SGCs (Fig. 3A, B). Thus experiments were carried out to examine whether an ectopic expression of *CYCA2;3* under the control of the *FAMA* promoter has an impact on GMC divisions. Although SGCs were still occasionally found, the defect of GMC division in *35S:CDKB1;1.N161* plants was overcome by expression of *FAMA:CYCA2;3* (Table 1; Fig. 3D). Strikingly, expression of *FAMA:CYCA2;3* in wild-type plants produced stomata with 3–4 guard cells (Fig. 3C), indicating that elevated levels of *CYCA2;3* sustain divisions in the GCs after the GMC symmetric division.

**Fig. 5.**
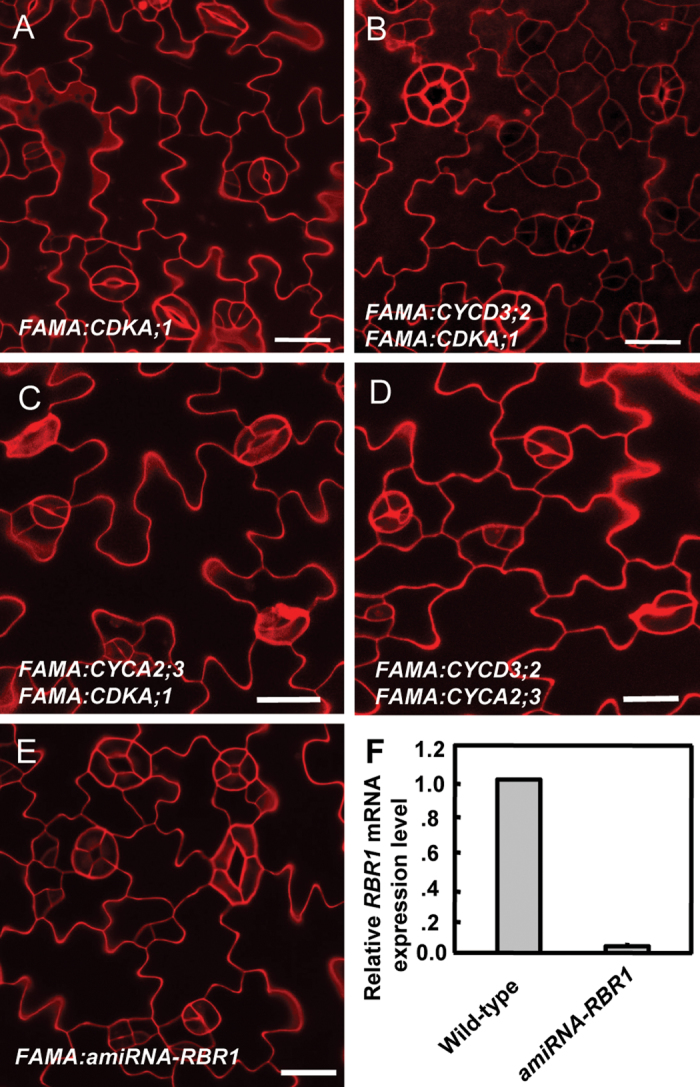
The synergistic effect of co-expression of *CYCD3;2* and *CDKA;1* in promoting GC subdivisions requires RBR1. (A) No obvious stomatal defect was detected in *FAMA:CDKA;1*. (B) Co-expression of *FAMA:CDKA;1* with *FAMA:CYCD3;2* augments GC subdivisions. (C, D) Co-expression of *FAMA:CYCA2;3* with either *FAMA:CYCD3;2* or *FAMA:CDKA;1* mimics stomatal phenotypes in *FAMA:CYCA2;3.* (E) GC subdivisions in *FAMA:amiRNA-RBR1*. (F) *RBR1* transcription is significantly suppressed in a *FAMA:amiRNA-RBR1* line. Bars=20 μm. (This figure is available in colour at *JXB* online.)

Moreover expression of each member gene of the *CYCD3* family under the control of the *FAMA* promoter induced three- or four-celled stomata as well (Fig. 3E; Supplementary Fig. S2 at *JXB* online). No SGC was found in *35S:CDKB1;1.N161* plants harbouring *FAMA:CYCD3;2*, indicating that increasing levels of D-type cyclin can reverse the G_2_ arrest in these dominant-negative *CDKB1;1.N161* transgenic plants (Fig. 3F). This suggested that sufficient CDK activity still persists in the GC after the terminal division, but that the levels of A- or D-type cyclins become rate limiting for another round of division (Table 1). RT-qPCR assays revealed that *CDKA;1* transcript levels normalized against the transcripts of the GC-specific *KAT1* gene (Xie *et al.*, 2010) were up-regulated in both *FAMA:CYCA2;3* and *FAMA:CYCD3;2* transgenic plants, suggesting that an enhancement in *CDKA;1* expression is associated with *CYCA2;3*- or *CYCD3;2*-promoted cell proliferation (Fig. 3G). However, given that *FAMA:CDKA;1* (Fig. 5A) is not sufficient to induce stomata with extra GCs, the level of cyclins might become rate limiting for divisions in the GC.

### Elevating CYCA2;3 levels in GC causes cellular fate reversion in GCs

**Fig. 4.**
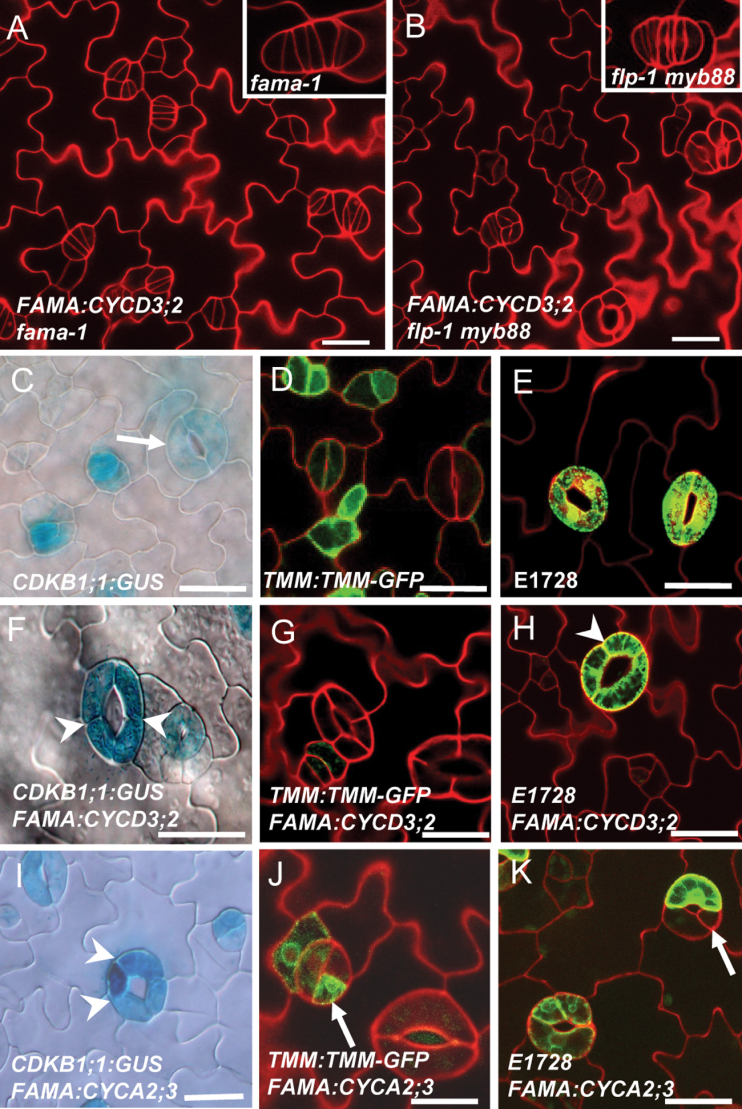
Ectopic expression of *CYCD3;2* or *CYA2;3* induces GC subdivisions. (A) The loss of *FAMA* function phenotype is epistatic to *FAMA:CYCD3;2*, presumably because *fama-1* blocks the acquisition of GC fate. Inset: a *fama-1* tumour. (B) *FAMA:CYCD3;2* and *flp-1 myb88* together show additive phenotypes of stomatal clusters and GC subdivisions. Inset: *flp-1 myb88* stomatal cluster. (C) *CDKB1;1:GUS,* a marker for the stomatal terminal differentiation stage, expressed from the transition stage of GMC to GC in the wild type. Note the weak GUS expression in a mature stoma (an arrow). (D) *TMM:TMM-GFP* expression in stomatal lineage cells in the wild type. (E) E1728, a mature GC marker expressed in wild-type GCs. (F) Equally enhanced *CDKB1;1:GUS* expression in a subdivided *FAMA:CYCD3;2* stoma. Arrowheads point to the extra cell division planes. (G) No *TMM:TMM-GFP* expression in *FAMA:CYCD3;2*-subdivided GCs. (H) Subdivided GCs induced by *FAMA:CYCD3;2* eventually exhibit a GC fate as shown by the expression of the E1728 marker. An arrow points to the subdivision plane. (I) Expression of *CDKB1;1:GUS* in a stoma with four GCs of *FAMA:CYCA2;3*. An arrow points to the GC showing a differential higher *GUS* staining than its sister cell. (J) Ectopic expression of *TMM:TMM-GFP* could be found in one of the subdivided GCs, indicated by an arrow. (K) *FAMA*:*CYCA2;3*-induced GC subdivision mimics *FAMA:CYCD3;2*, but some of them were unequal divisions. E1728 GFP fluorescence is from a mature GC but was absent in another GC (arrow). Bars=20 μm. (This figure is available in colour at *JXB* online.)

FAMA is also required for GMC to GC differentiation to acquire GC fate. Loss of *FAMA* function leads to narrow cell tumours that lack GC fate. *FAMA:CYCD3;2* was crossed into a *fama-1* null mutant. Elevating *CYCD3* levels did not trigger a synergistic effect on cell proliferation in the *fama-1* mutant background (Fig. 4A). In contrast, *FAMA:CYCD3;2* in the *flp-1 myb88* double mutant background, in which some cells of the stomatal clusters could acquire GC fate, produced perpendicular as well as parallel divisions, an additive phenotype consistent with *FAMA:CYCD3;2* inducing GC subdivisions after GMC division (Fig. 4B).

Expression of *CDKB1;1:GUS* is found in GMCs and young GCs, but is low in mature GCs (Fig. 4C). Overexpression of *FAMA:CYCD3;2* induced an enhanced expression of *CDKB1;1:GUS* in subdivided GCs. However, the equal *GUS* expression levels among all subdivided GCs indicate that these divisions are symmetric (Fig. 4F). *TMM:TMM-GFP* (green fluorescent protein) is a stomatal lineage marker expressed in GMCs, young GCs, and stomatal lineage ground cells; but it is absent in mature guard cells (Fig. 4D). The absence of strong *TMM:TMM-GFP* expression in *FAMA:CYCD3;2* subdividing GCs suggests that these extra divisions occurred at the final stage of stomatal development (Fig. 4G). Moreover, like the wild type, all subdivided GCs eventually express the mature GC marker E1728 (Fig. 4E, H), indicating that *CYCD3;2*-induced extra divisions are indeed symmetric divisions in GC and uncoupled from GC differentiation.

Overexpression of *CYCA2;3* at the stage of *FAMA* expression caused a dramatic increase in the *CYCA2;3* transcription level (Supplementary Fig. S3A at *JXB* online). Expression of *FAMA:CYCA2;3* also induced a differential increasing expression of *CDKB1;1:GUS* in some subdivided GCs, which was not observed in *FAMA:CYCD3;2* lines (arrow in Fig. 4I versus F). Strikingly, ectopic high expression of *TMM:TMM-GFP* was found in some subdivided cells in *FAMA:CYCA2;3* stomata, implying a cell fate reversion from a GC back to a precursor cell or at least to an immature GC (Fig. 4J). Consistently, the GC marker E1728 is not always expressed evenly in the different subdivided cells within the same stoma, implying that their GC differentiation was disturbed (Fig. 4K). In addition, collapsed cells were often found in *FAMA:CYCA2;3* stomata, which are not found in *FAMA:CYCD3;2*, confirmed by a cell viability assay using Neutral Red dyes (Supplementary Figs S3B–E, S4 at JXB online).

### CDKA;1–CYCD3;2 complexes stimulate extra symmetric GC subdivisions

**Fig. 6.**
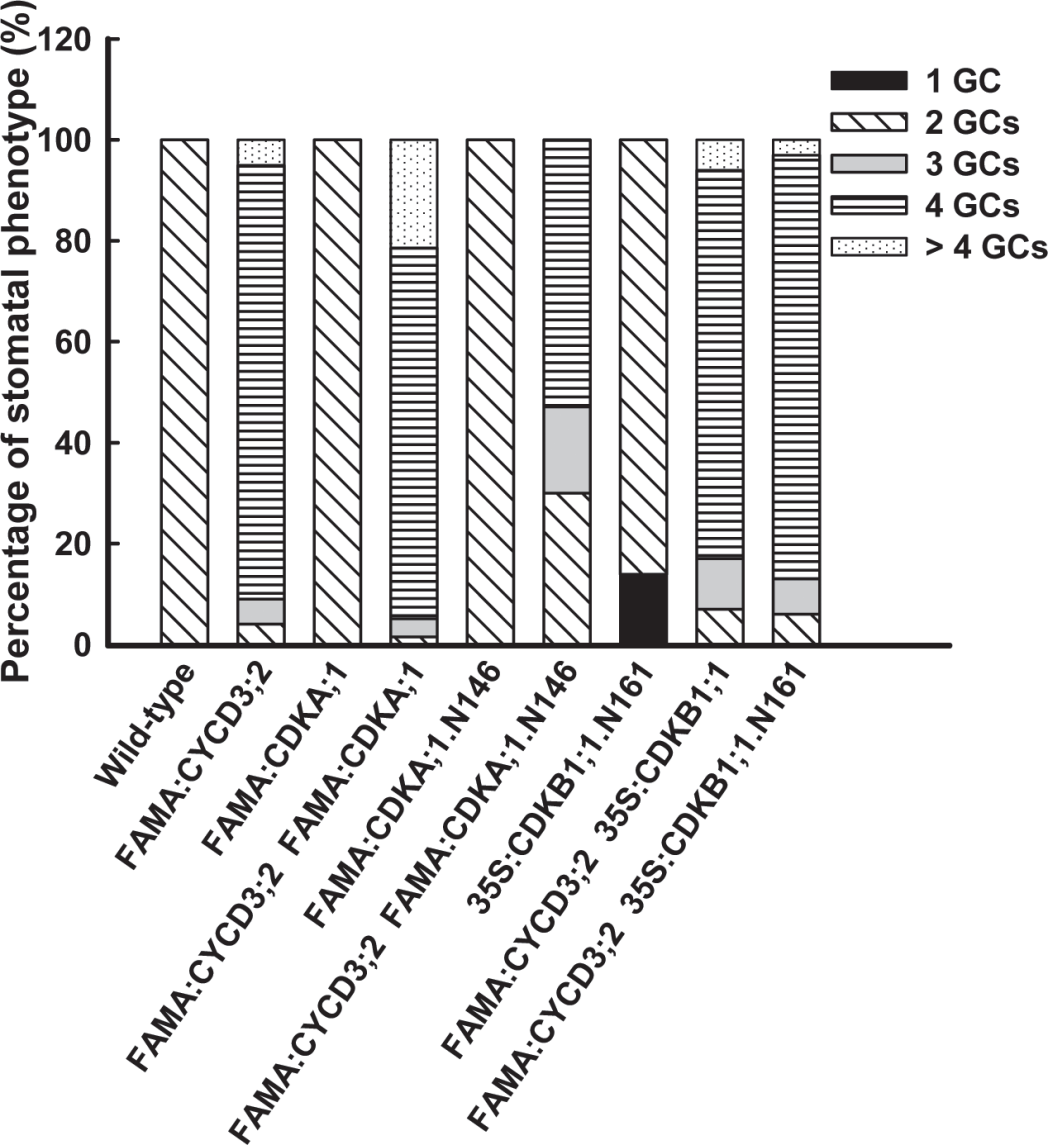
Quantification of effects of different CDK forms on *FAMA:CYCD3;2* stomatal phenotypes. Quantification of stomatal phenotypes from 14-day-old cotyledons. A total of 800–1000 stomata from 20–30 cotyledons were scored for each genotype. Black, SGCs; oblique, normal stomata with two GCs; grey, stomata with three GCs; hatched, stomata with four GCs; dotted, stomata with >4 GCs.

To probe the genetic relationship between *CDKA;1* and *CYCD3;2* during stomatal development, targeted *CYCD3;2* and *CDKA;1* overexpression was initially introduced individually into wild-type plants under the respective control of the *SPCH*, *MUTE*, and *FAMA* promoters. Subsequently, lines with *CYCD3;2* and *CDKA;1* driven by the same promoter were crossed. No obvious stomatal defects were found in plants harbouring both *SPCH:CDKA;1* and *SPCH:CYCD3;2*, or in those containing *MUTE:CDKA;1* and *MUTE:CYCD3;2* (Supplementary Fig. S5A, B at *JXB* online). Although stomatal development is normal in *FAMA:CDKA;1* transformants (Fig. 5A), co-expression of *FAMA:CDKA;1* with *FAMA:CYCD3;2* greatly enhanced the GC subdivision phenotype (Fig. 5B; Supplementary Fig. S5C at *JXB* online). In *FAMA:CYCD3;2* plants, most stomata (90%) contained 3–4 GCs, while 5% were normal and 5% had >4 GCs. The co-expression of both *FAMA:CYCD3;2* and *FAMA:CDKA;1* in wild-type plants increased the fraction of stomata containing >4 GCs to 21% and the percentage of normal stomata was reduced to 1% (Fig. 6).

In contrast, the co-expression of a dominant-negative version of *FAMA:CDKA;1.N146* with *FAMA:CYCD3;*2 increased the fraction of normal stomata to ~30% and decreased the number of stomata containing >4 GCs (Fig. 6). However, CDKB1;1 activity is not required for CYCD3;2’s functions, since co-expression with either *FAMA:CDKB1;1* or *35S:CDKB1;1.N161* has no impact on *FAMA:CYCD3;2*-induced GC subdivisions (Fig. 6). The above synergistic effects on GC subdivisions were not found between *CYCA2;3* and *CDKA;1*, or between *CYCA2;3* and *CYCD3;2* (Fig. 5C, D; Supplementary Fig. S4 at *JXB* online).

The potential for CYCD3;2 to interact directly with CDKA;1 was demonstrated in yeast two-hybrid assays and BiFC *in vivo* assays (Supplementary Fig. S5D, E at *JXB* online). Furthermore, a pull-down experiment further proved that CYCD3;2 forms a protein complex with CDKA;1 *in vitro* (Supplementary Fig. S5F at *JXB* online).

### Suppression of *RBR1* also induced GC subdivisions

*FAMA*-driven expression of *CYCA2;3* or *CYCD3;2* induced 3–4 cells radially arranged around the pore. This stomatal phenotype resembles those stomata found in an inducible RNAi against *RBR1* plants (Borghi *et al.*, 2010). Development of pavement cells and stomatal formation are also disrupted by the loss of RBR1 function, consistent with its wide expression in the epidermis (Supplementary Fig. S6A at *JXB* online). GC subdivison is observed in a line of artificial microRNA (*amiRNA*) against *RBR1* under the *FAMA* promoter, *FAMA:amiRNA-RBR1*, in which *RBR1* transcription is significantly suppressed (Fig. 5E, F).

The *RBR1:GUS* expression pattern and RT-qPCR assays reveal that the impact of *FAMA:CYCD3;2* on *RBR1* transcription is limited (Supplementary S6B, C at *JXB* online). DAPI staining analysis demonstrates that the DNA ploidy levels in the subdivided GCs found in *FAMA:amiRNA-RBR1* and *FAMA:CYCD3;2* epidermis are the same as in normal GCs, indicating that ectopic expression of *CYCD3;2* or suppression of *RBR1* promotes cell proliferation only (Supplementary Fig. S6D–J).

Most of the stomata in *FAMA:CYCD3;2 FAMA:CDKA;1* consist of 3–4 GCs (78%), while 21% of stomata have >4 GCs. Each daughter GC has its own cell outline and contacts with the adjacent GCs, displaying a ‘string of sausages’-like stoma (Type II in Supplementary Fig. S7 at *JXB* online). In one of the *FAMA:amiRNA-RBR1* lines, 25.7% stomata mimic the ‘string of sausages’-like stomata. Around 30% of stomata in this *FAMA:amiRNA-RBR1* line exhibit ectopic divisions in their GCs. However, these extra divisions happened in original GCs (produced by GMC division) as well, but did not alter the original GC shape (Type III in Supplementary Fig. S7).

## Discussion

### CDKA;1 activity is required for GMC division

Whereas in yeast a single CDKA;1 regulates both the G_1_ to S and G_2_ to M transition of the mitotic cell cycle, the CDKB kinases, together with CDKA;1 kinase complexes, operate at the G_2_ to M transition in *Arabidopsis* plants (Menges *et al.*, 2005). *CDKA;1* has pleiotropic functions in plant development, including the maintenance of shoot and root apical meristems (Dissmeyer *et al.*, 2009; Nowack *et al.*, 2012). Mutations in *CDKA;1* induce a failure in the double fertilization and lead to the abortion of homozygous *cdka;1* seeds. Homozygous *cdka;1* mutants recovered using a *PRO*_*CDKA;1*_ *:CDKA;1:YFP* construct exhibit defects in embryogenesis and in the maintenance of the root and shoot apical meristems (Nowack *et al.*, 2012). Homozygous *cdka;1* seedlings were recovered via a pollen-specific promoter to drive *CDKA;1* gene expression (*LAT52:CDKA:1*). These *cdka;1* homozygous seedlings showed significant reduction in stomatal formation and undivided GMCs, indicating that CDKA;1 activity is required for both stomatal formative asymmetric divisions and terminal symmetric divisions, consistent with the expression of *CDKA;1* throughout the stomatal lineage cells (Serna and Fenoll, 1997). Using a dominant-negative *CDKA;1.N146* construct driven by either the *MUTE* or the *FAMA* promoter, it was possible to dissect further the role of *CDKA;1* in stomatal development. Triggering the expression of dominant-negative *CDKA;1.N146* with the *MUTE* promoter impaired the GMC symmetric division in forming the two GCs, whereas later expression of *CDKA;1.N146* with the *FAMA* promoter hardly influenced the symmetric division, indicating that the timing of CDKA;1 activity plays an important role in the early events corresponding to the specification of the GMC. However, even in these GMCs with reduced CDKA;1 activity, the cell cycle can be uncoupled from GMC to GC differentiation as the undivided GMC arrested early in the cell cycle can eventually acquire mature GC fate, in line with the GC differentiation upon interfering with CDKB1 activity (Xie *et al.*, 2010).

**Fig. 7.**
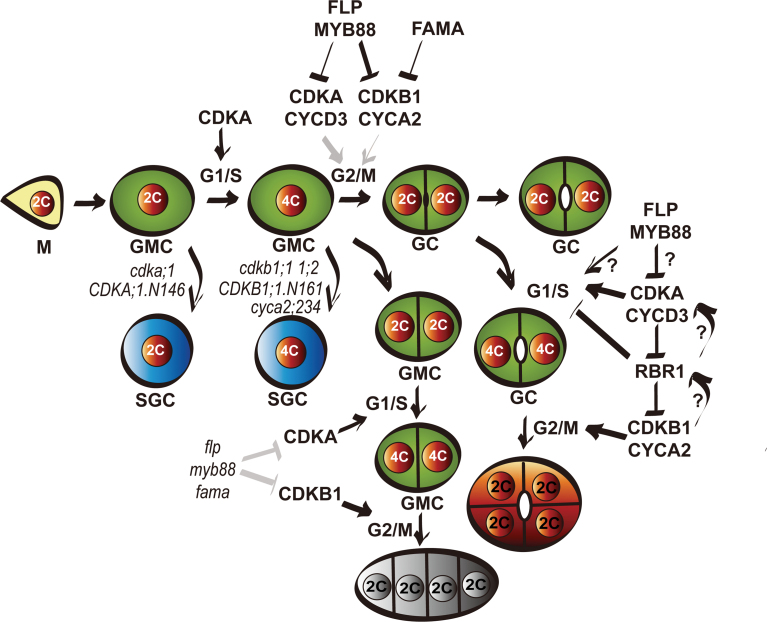
Regulatory network of terminal division of stomatal development. When the meristemoid (M) acquires a GMC fate, CDKA;1 activity is required for G_1_ to S transition in the cell cycle. In the loss-of-function *cdka;1* mutant or dominant-negative *CDKA;1.N146* transgenic lines, an undivided GMC still could acquire a GC fate, resulting in SGCs with 2C ploidy. *CDKB1* and *CYCA2* are required for the G_2_ to M transition phase. In the *cdkb1;1 1;2* double mutant or dominant-negative *CDKB1;1.N161* lines, the undivided GMCs differentiate into SGCs with 4C ploidy. Mutants of *cyca2;234* also produce SGCs with 4C ploidy. *CDKA;1, CDKB1;1*, and *CYCA2* transcription can all be negatively regulated by FLP/MYB88 transcriptional factors through *cis*-regulatory elements. Expression of *CDKA;1* could partially rescue GMC division defects. Overexpression of *CYCA2* or *CYCD3* also reduces the number of SGCs in *CDKB1;1.N161*. In *flp myb88* and *fama* mutants, high activity of CDKA;1 and CDKB1 causes excessive GMC divisions. RBR1 is essential to prevent GC subdivision, but the regulatory network of CDK–cyclin–RBR1 and the feedback loop in terminal division remain unclear. (This figure is available in colour at *JXB* online.)

Abnormal SGCs were first reported in *35S:CDKB1;1.N161*, then in c*dkb1;1 1;2* double mutants, and in *cyca2;234* triple mutants (Boudolf *et al.*, 2004*a*; Xie *et al.*, 2010; Vanneste *et al.*, 2011). Despite their similar cell size, SGCs in loss-of-function *cdka;1* homozygous mutants or targeted expression of dominant-negative *CDKA;1.N146* lines contain nuclear DNA levels comparable with those of GCs with 2C DNA levels in wild-type stomata, suggesting that *CDKA;1* acts before S-phase at the G_1_ to S transition of the cell cycle (Fig. 7). In contrast, SGCs produced in *cdkb1* or *cyca2* mutants display twice the DNA content of wild-type GCs, as a result of cell cycle arrested at the G_2_ to M transition (Boudolf *et al.*, 2004*a*; Xie *et al.*, 2010; Vanneste *et al.*, 2011).

Overexpression of *CDKA;1* under control of *FAMA* at the same stage could partially rescue the GMC division in the *cdkb1;1 1;2* mutant, suggesting that elevated CDKA;1 kinase activity can, at least partially, substitute for CDKB1 activity. Either CDKA;1 or CDKB1 kinases can phosphorylate the same substrates, albeit with a different efficiency, for cell cycle progression. Alternatively, elevating the CDKA;1 level might reduce the threshold for the overall CDKB1 kinase activity required for G_2_ to M transition. In line with this observation, expression of *CDKA;1* driven by the *TMM* promoter displayed a similar rescuing effect (Weimer *et al.*, 2012). Although CDKA;1 activity is generally more important for the G_1_ to S transition and CDKB1s are required for G_2_ to M progression (Boudolf *et al.*, 2004*a*), the present results indicate that the mechanism for G_2_ to M transition has some degree of flexibility, and that in the absence of CDKB1, elevated CDKA;1 can trigger the G_2_ to M transition.

### CDKA;1 could be a direct target of FLP/MYB88

A model has been proposed for the function of *FLP/MYB88* in stomatal development, in which *FLP/MYB88* enforce cell cycle exit after GMC division by timely suppression of cell cycle genes for further divisions. This is supported by the expression pattern of the promoter reporters, such as *CDKB1;1:GFP* and *CYCA2;3:GUS-GFP* which are expressed in late GMCs and young GCs, similar to the expression pattern of *FLP/MYB88* or *FAMA* (Xie *et al.*, 2010; Vanneste *et al.*, 2011). Using a *CDKA;1* transcriptional reporter included a fusion of yellow fluorescent protein (YFP) with a cyclin B destruction box (DB), *ProCDKA;1:YFP-DB*, *CDKA;1* expression appears to shut off immediately after GMC division (Xie *et al.*, 2010). Up-regulation of *CDKA;1* and *CDKB1;1* transcript levels was found in *flp-1 myb88* mutants. Hence it seems that *CDKA;1* and the earlier reported *CDKB1* genes (Xie *et al.*, 2010) are both involved in the GMC divisions and regulated by FLP/MYB88 transcription factors (Fig. 7).

*FLP* and *MYB88* genes encode an atypical two-MYB-repeat transcription factor with binding preferences different from those of other known MYBs. However, FLP/MYB88 binds to the *CDKB1;1* promoter via the [A/T/G][A/T/G]C[C/G][C/G] consensus sequence, a *cis*-regulatory element overlapping with that of cell cycle transcription factor E2Fa (Boudolf *et al.*, 2004*b*; Xie *et al.*, 2010). Here four *cis*-regulatory elements in the *CDKA;1* promoter for FLP/MYB88 binding were identified, suggesting that *CDKA;1*, like *CDKB1;1*, is directly regulated by the FLP/MYB88 transcription factors, as part of the mechanism to restrict symmetric divisions to a single event.

### Elevating cyclin A or D confers ectopic cell cycle activation in GC

Elevating *CDKA;1* in the GMC with the *pFAMA:CDKA;1* construct (bypassing the FLP/MYB88 control) was not sufficient to confer additional divisions, raising the question of whether cyclin levels were rate limiting for ectopic cell cycle activation. Constitutive *CYCD3;1* expression alters leaf epidermal cell proliferation and stomatal density, but does not induce morphological defects in stomatal development (Dewitte and Murray, 2003; Dewitte *et al.*, 2007; Elsner *et al.*, 2012). However, the targeted expression of *CYCD3;1* in normally endocyling trichomes by the *GLABRA2* promoter triggers mitosis, leading to subdivided trichomes, a phenotype not observed in *35S:CYCD3;1* plants (Schellmann *et al.*, 2002; Dewitte *et al.*, 2003). A similar difference between targeted and constitutive expression of *CYCD3* genes was observed in stomatal development. Whereas constitutive *CYCD3;1* expression does not interfere with the GCs and allows for cell cycle arrest, the *FAMA* promoter probably increases local *CYCD3;2* levels sufficiently to induce GC subdivisions, a gain-of-function phenotype, like *GL2:CYCD3;1* in trichomes.

A similar phenotype was observed when *CYCA2;3* levels were increased during and after the last division. However, in the case of *CYCA2;3*, the formed GC occasionally reverted to a precursor state or caused cell collapse. Indeed, an extremely high transcript level of C*DKA;1* was observed in *FAMA:CYCA2;3* stomata, but it is not clear whether the programmed cell death was stimulated by altered levels of CDKA;1 or RBR1, a regulation model proposed in trichome and endosperm development (Schnittger *et al.*, 2003; Sabelli *et al.*, 2013). Hence, cyclins can confer ectopic cell cycle activation, suggesting that the maintenance of two-celled stomata requires accurate control of cyclin A and D levels.

### Cyclin A and D require different CDKs in order to stimulate GC division

Although the *cdkb1;1* single mutant displays normal stomata, *cdkb1;1 cyca2;234* quadruple mutants show more SGCs than *cyca2;234* triple mutants, suggesting that CYCA2s and CDKB1;1 contribute synergistically to the CDK activity required for GMC division (Vanneste *et al.*, 2011). Co-expressing *CDKB1;1* with *CYCA2;3* could enhance CYCA2;3-associated kinase activity, demonstrating that CYCA2;3 and CDKB1;1 form a functional complex stimulating cell divisions. Introduction of *CYCA2;3* driven by the 35S promoter could complement the endoreduplication phenotype of *35S:CDKB1;1.N161* plants, but could not rescue its GMC division defects (Boudolf *et al.*, 2009). Here the targeted expression of *CYCA2;3* has little effect when CDKB1;1 function is impaired by overexpression of dominant-negative *CDKB1;1.N161*. Although overexpression of either *CYCA2;3* or *CYCD3;2* was associated with up-regulation of the *CDKA;1* transcript, it appears that only CYCD3;2/CDKA;1, and not CYCA2;3/CDKA;1. is able to stimulate the G_2_ to M transition (Healy *et al.*, 2001; Imai *et al.*, 2006). The findings show that the CYCD3;2 and CDKA;1 proteins can interact directly. The extensive subdivision of GCs induced by transformation with *FAMA:CYCD3;2* can in turn be partially repressed by the concomitant expression of the dominant-negative *CDKA;1.N146* construct. In addition, the degree of GC subdivision is profoundly enhanced by co-expression of *CDKA;1*. The gain or loss of function of *CDKB1* genes (via the co-expression of *CDKB1;1* or the dominant-negative *CDKB1;1.N161* construct) does not affect the extent of *FAMA:CYCD3;2*-induced GC subdivision, suggesting that the functions of CDKA;1 and CDKB1s differ with respect to their cooperation with CYCD3;2.

### RBR1 restricts GC proliferation

Arabidopsis possess only a single *RBR1* gene modulating cell division and endoreduplication (Gutzat *et al.*, 2012). Cell cycle progression is regulated through RBR1 protein phosphorylation by G_1_/S kinases such as CYCD–CDKA;1 (Nakagami *et al.*, 2002). Indeed the function of CDKA;1 in stomatal asymmetric division was found to be mediated by RBR1 (Nowack *et al.*, 2012). Genes required for stomata initiation, such as *TMM* and *SPCH*, were up-regulated in *RBRi* plants, but expression of *MUTE* and *FAMA* was not affected (Borghi *et al.*, 2010). However, similar to overexpression of *CYCD3;2* or *CYCA2;3*, suppression of *RBR1* in GMCs and GCs with the *FAMA:amiRNA-RBR1* caused extra division in GCs but not in GMCs, the subdividing GCs being a phenotype distinct from the narrow parallel arranged small cells in *flp myb88* or *fama* mutants. Consistent with this, overexpressing *FAMA:CYCD3;2* in *fama-1* has no influence on the size of cell tumours. The RBR1 level is regulated appropriately in wild-type GCs to ensure GCs do not re-enter the cell cycle and maintain GC integrity. Thus, besides a role in stomatal initiation, *RBR1* functions prevent GC overproliferation during the last stage of stomatal formation.

In summary, over-riding the control mechanisms involved in cell cycle arrest in the last phase of GC development by elevating levels of CYCD/CDKA;1, CYCA/CDKB kinases, or alternatively by suppressing the *RBR1* level sustains cell proliferation in the stomatal lineage, resulting in GC subdivisions. However, since both CDKA;1 and CDKB;1 activity are required for proper execution of the terminal cell cycle, timely suppression of *CDKA;1* expression by FLP/MYB is likely to be contributing to the final cell cycle arrest in mature stomata (Fig. 7).

## Supplementary data

Supplementary data are available at *JXB* online.

Figure S1. EMSA results show that MYB88 can bind the *CDKA;1* promoter.

Figure S2. Expression of *CYCD3;1* or *CYCD3;3* promotes GC subdivisions.

Figure S3. Overexpression of *CYCA2;3* induces GC subdivision and cell collapse.

Figure S4. Quantification of the effect of co-expression of *CYCA2;3* and *CDKB1;1* in promoting GC subdivisions.

Figure S5. CYCD3;2 directly interacts with CDKA;1.

Figure S6. *RBR1* expression and GC subdivision.

Figure S7. Comparision of stomatal phenotypes between *CYCD3;2 CDKA;1* co-expression and *RBR1 RNAi* lines.

Table S1. Primer sequences used in this article.

Supplementary Data

## Abbreviations:

CDKA: 1, CYCLIN DEPENDENT KINASE A1
CYCD3: CYCLIN D3
CYCA2: CYCLIN A2
DAPI: 4′,6-diamidino-2-phenylindole
FLP: FOUR LIPS
GC: guard cell
GMC: guard mother cell
MMC: meristemoid mother cell
RBR1: RETINOBLASTOMA-RELATED 1
SGC: single guard cell.

## References

[CIT0001] Bergmann DC Sack FD 2007 Stomatal development. Annual Review of Plant Biology 58, 163–181 10.1146/annurev.arplant.58.032806.104023 17201685

[CIT0002] Borghi L Gutzat R Futterer J Laizet YH Hennig L Gruissem W 2010 Arabidopsis RETINOBLASTOMA-RELATED is required for stem cell maintenance, cell differentiation, and lateral organ production. The Plant Cell 22, 1792–1811 20525851 10.1105/tpc.110.074591 PMC2910961

[CIT0003] Boudolf V Barroco R Engler Jde A Verkest A Beeckman T Naudts M Inze D De Veylder L 2004a B1-type cyclin-dependent kinases are essential for the formation of stomatal complexes in *Arabidopsis thaliana* . The Plant Cell 16, 945–955 15031414 10.1105/tpc.021774 PMC412868

[CIT0004] Boudolf V Lammens T Boruc J 2009 CDKB1;1 forms a functional complex with CYCA2;3 to suppress endocycle onset. Plant Physiology 150, 1482–1493 19458112 10.1104/pp.109.140269 PMC2705057

[CIT0005] Boudolf V Vlieghe K Beemster GTS Magyar Z Acosta JAT Maes S Van Der Schueren E Inzé D De Veylder L 2004b The plant-specific cyclin-dependent kinase CDKB1;1 and transcription factor E2Fa-DPa control the balance of mitotically dividing and endoreduplicating cells in Arabidopsis. The Plant Cell 16, 2683–2692 15377755 10.1105/tpc.104.024398 PMC520964

[CIT0006] Cruz-Ramirez A Diaz-Trivino S Blilou I 2012 A bistable circuit involving SCARECROW–RETINOBLASTOMA integrates cues to inform asymmetric stem cell division. Cell 150, 1002–1015 22921914 10.1016/j.cell.2012.07.017 PMC3500399

[CIT0007] De Veylder L Beeckman T Van Montagu M Inzé D 2000 Increased leakiness of the tetracycline-inducible Triple-Op promoter in dividing cells renders it unsuitable for high inducible levels of a dominant negative CDC2aAt gene. Journal of Experimental Botany 51, 1647–1653 11053453 10.1093/jexbot/51.351.1647

[CIT0008] Dewitte W Murray JAH 2003 The plant cell cycle. Annual Review of Plant Biology 54, 235–264 10.1146/annurev.arplant.54.031902.134836 14502991

[CIT0009] Dewitte W Scofield S Alcasabas AA 2007 Arabidopsis CYCD3 D-type cyclins link cell proliferation and endocycles and are rate-limiting for cytokinin responses. Proceedings of the National Academy of Sciences, USA 104, 14537–14542 10.1073/pnas.0704166104 PMC1964848 17726100

[CIT0010] Dissmeyer N Weimer AK Pusch S 2009 Control of cell proliferation, organ growth, and DNA damage response operate independently of dephosphorylation of the Arabidopsis Cdk1 homolog CDKA;1. The Plant Cell 21, 3641–3654 19948791 10.1105/tpc.109.070417 PMC2798325

[CIT0011] Elsner J Michalski M Kwiatkowska D 2012 Spatiotemporal variation of leaf epidermal cell growth: a quantitative analysis of Arabidopsis thaliana wild-type and triple cyclinD3 mutant plants. Annals of Botany 109, 897–910 22307569 10.1093/aob/mcs005 PMC3310487

[CIT0012] Gaamouche T Manes C-LdO Kwiatkowska D 2010 Cyclin-dependent kinase activity maintains the shoot apical meristem cells in an undifferentiated state. The Plant Journal 64, 26–37 20659279 10.1111/j.1365-313X.2010.04317.x

[CIT0013] Gardner MJ Baker AJ Assie J-M Poethig RS Haseloff JP Webb AAR 2009 GAL4 GFP enhancer trap lines for analysis of stomatal guard cell development and gene expression. Journal of Experimental Botany 60, 213–226 19033548 10.1093/jxb/ern292 PMC3071773

[CIT0014] Gutierrez C 2008 Coordination of cell division and differentiation. In: Verma DPS Hong Z, eds. *Cell division control in plants*. Heidelberg: Springer-Verlag, 377–393

[CIT0015] Gutzat R Borghi L Gruissem W 2012 Emerging roles of RETINOBLASTOMA-RELATED proteins in evolution and plant development. Trends in Plant Science 17, 139–148 22240181 10.1016/j.tplants.2011.12.001

[CIT0016] Harbour JW Dean DC 2000 Chromatin remodeling and Rb activity. Current Opinion in Cell Biology 12, 685–689 11063932 10.1016/s0955-0674(00)00152-6

[CIT0017] Healy JMS Menges M Doonan JH Murray JAH 2001 The Arabidopsis D-type cyclins CycD2 and CycD3 both interact *in vivo* with the PSTAIRE cyclin-dependent kinase Cdc2a but are differentially controlled. Journal of Biological Chemistry 276, 7041–7047 11096103 10.1074/jbc.M009074200

[CIT0018] Hemerly A de Almeida Engler J Bergounioux C Van Montagu M Engler G Inze D Ferreira P 1995 Dominant negative mutants of the Cdc2 kinase uncouple cell division from iterative plant development. EMBO Journal 14, 3925–3936 7664733 10.1002/j.1460-2075.1995.tb00064.x PMC394471

[CIT0019] Imai KK Ohashi Y Tsuge T Yoshizumi T Matsui M Oka A Aoyama T 2006 The A-type cyclin CYCA2;3 is a key regulator of ploidy levels in Arabidopsis endoreduplication. The Plant Cell 18, 382–396 16415207 10.1105/tpc.105.037309 PMC1356546

[CIT0020] Inze D De Veylder L 2006 Cell cycle regulation in plant development. Annual Review of Genetics 40, 77–105 10.1146/annurev.genet.40.110405.090431 17094738

[CIT0021] Iwakawa H Shinmyo A and Sekine M 2006 Arabidopsis CDKA;1, a cdc2 homologue, controls proliferation of generative cells in male gametogenesis. The Plant Journal 45, 819–831 16460514 10.1111/j.1365-313X.2005.02643.x

[CIT0022] Joubès J De Schutter K Verkest A Inzé D De Veylder L 2004 Conditional, recombinase-mediated expression of genes in plant cell cultures. The Plant Journal 37, 889–896 14996220 10.1111/j.1365-313x.2004.02004.x

[CIT0023] Lai L Nadeau JA Lucas J Lee E-K Nakagawa T Zhao L Geisler MJ Sack FD 2005 The Arabidopsis R2R3 MYB proteins FOUR LIPS and MYB88 restrict divisions late in the stomatal cell lineage. The Plant Cell 17, 2754–2767 16155180 10.1105/tpc.105.034116 PMC1242270

[CIT0024] Lau OS Bergmann DC 2012 Stomatal development: a plant’s perspective on cell polarity, cell fate transitions and intercellular communication. Development 139, 3683–3692 22991435 10.1242/dev.080523 PMC3445305

[CIT0025] Lee E Liu X Eglit Y Sack F 2013 FOUR LIPS and MYB88 conditionally restrict the G1/S transition during stomatal formation. Journal of Experimental Botany 64, 5207–5219 24123248 10.1093/jxb/ert313 PMC3830495

[CIT0026] MacAlister CA Ohashi-Ito K Bergmann DC 2007 Transcription factor control of asymmetric cell divisions that establish the stomatal lineage. Nature 445, 537–540 17183265 10.1038/nature05491

[CIT0027] Menges M De Jager SM Gruissem W Murray JAH 2005 Global analysis of the core cell cycle regulators of Arabidopsis identifies novel genes, reveals multiple and highly specific profiles of expression and provides a coherent model for plant cell cycle control. The Plant Journal 41, 546–566 15686519 10.1111/j.1365-313X.2004.02319.x

[CIT0028] Nadeau JA Sack FD 2002 Stomatal development in Arabidopsis. The Arabidopsis Book, e0066 22303215 10.1199/tab.0066 PMC3243354

[CIT0029] Nakagami H Kawamura K Sugisaka K Sekine M Shinmyo A 2002 Phosphorylation of retinoblastoma-related protein by the cyclin D/cyclin-dependent kinase complex is activated at the G1/S-phase transition in tobacco. The Plant Cell 14, 1847–1857 12172026 10.1105/tpc.002550 PMC151469

[CIT0030] Nowack MK Harashima H Dissmeyer N Zhao XA Bouyer D Weimer AK De Winter F Yang F Schnittger A 2012 Genetic framework of cyclin-dependent kinase function in Arabidopsis. Developmental Cell 22, 1030–1040 22595674 10.1016/j.devcel.2012.02.015

[CIT0031] Ohashi-Ito K Bergmann DC 2006 Arabidopsis FAMA controls the final proliferation/differentiation switch during stomatal development. The Plant Cell 18, 2493–2505 17088607 10.1105/tpc.106.046136 PMC1626605

[CIT0032] Pillitteri LJ Sloan DB Bogenschutz NL Torii KU 2007 Termination of asymmetric cell division and differentiation of stomata. Nature 445, 501–505 17183267 10.1038/nature05467

[CIT0033] Pillitteri LJ Torii KU 2012 Mechanisms of stomatal development. Annual Review of Plant Biology 63, 591–614 10.1146/annurev-arplant-042811-105451 22404473

[CIT0034] Porter A 2008 Preventing DNA over-replication: a Cdk perspective. Cell Division 3, 3 18211690 10.1186/1747-1028-3-3 PMC2245919

[CIT0035] Sabelli PA Liu Y Dante RA 2013 Control of cell proliferation, endoreduplication, cell size, and cell death by the retinoblastoma-related pathway in maize endosperm. Proceedings of the National Academy of Sciences, USA 110, E1827–E1836 10.1073/pnas.1304903110 PMC3651506 23610440

[CIT0036] Schellmann S Schnittger A Kirik V Wada T Okada K Beermann A Thumfahrt J Jurgens G Hulskamp M 2002 TRIPTYCHON and CAPRICE mediate lateral inhibition during trichome and root hair patterning in Arabidopsis. EMBO Journal 21, 5036–5046 12356720 10.1093/emboj/cdf524 PMC129046

[CIT0037] Schnittger A Weinl C Bouyer D Schobinger U Hulskamp M 2003 Misexpression of the cyclin-dependent kinase inhibitor ICK1/KRP1 in single-celled *Arabidopsis* trichomes reduces endoreduplication and cell size and induces cell death. The Plant Cell 15, 303–315 12566574 10.1105/tpc.008342 PMC141203

[CIT0038] Serna L Fenoll C 1997 Tracing the ontogeny of stomatal clusters in Arabidopsis with molecular markers. The Plant Journal 12, 747–755 9375390 10.1046/j.1365-313x.1997.12040747.x

[CIT0039] Tang W Wang W Chen D Ji Q Jing Y Wang H Lin R 2012 Transposase-derived proteins FHY3/FAR1 interact with PHYTOCHROME-INTERACTING FACTOR1 to regulate chlorophyll biosynthesis by modulating HEMB1 during deetiolation in Arabidopsis. The Plant Cell 24, 1984–2000 22634759 10.1105/tpc.112.097022 PMC3442582

[CIT0040] Vanneste S Coppens F Lee E 2011 Developmental regulation of CYCA2s contributes to tissue-specific proliferation in Arabidopsis. EMBO Journal 30, 3430–3441 21772250 10.1038/emboj.2011.240 PMC3160660

[CIT0041] Walter M Chaban C Schütze K 2004 Visualization of protein interactions in living plant cells using bimolecular fluorescence complementation. The Plant Journal 40, 428–438 15469500 10.1111/j.1365-313X.2004.02219.x

[CIT0042] Weimer AK Nowack MK Bouyer D Zhao X Harashima H Naseer S De Winter F Dissmeyer N Geldner N Schnittger A 2012 Retinoblastoma related1 regulates asymmetric cell divisions in Arabidopsis. The Plant Cell 24, 4083–4095 23104828 10.1105/tpc.112.104620 PMC3517237

[CIT0043] Xie Z Lee E Lucas JR Morohashi K Li D Murray JAH Sack FD Grotewold E 2010 Regulation of cell proliferation in the stomatal lineage by the Arabidopsis MYB FOUR LIPS via direct targeting of core cell cycle genes. The Plant Cell 22, 2306–2321 20675570 10.1105/tpc.110.074609 PMC2929110

