# Supplementary material for: Requirement for A-type cyclin-dependent kinase and cyclins for the terminal division in the stomatal lineage of Arabidopsis
Source: J Exp Bot. 2014 Mar 31;65(9):2449–61. doi: 10.1093/jxb/eru139 (PMC4036514; doi:10.1093/jxb/eru139)
Supplement: Supplementary Data [file supp_65_9_2449__index.html]

Requirement for A-type cyclin-dependent kinase and cyclins for the terminal division in the stomatal lineage of Arabidopsis — Requirement for A-type cyclin-dependent kinase and cyclins for the terminal division in the stomatal lineage of Arabidopsis — Supplementary Data 

# Requirement for A-type cyclin-dependent kinase and cyclins for the terminal division in the stomatal lineage of *Arabidopsis*

## Supplementary Data

Data files

**Files in this Data Supplement:**

- Supplementary Data - Supplementary Data
