# Supplementary material for: Requirement for A-type cyclin-dependent kinase and cyclins for the terminal division in the stomatal lineage of Arabidopsis
Source: J Exp Bot. 2014 Mar 31;65(9):2449–61. doi: 10.1093/jxb/eru139 (PMC4036514; doi:10.1093/jxb/eru139)
Supplement: Supplementary Data [file supp_eru139_jexbot112391_file001.pdf]

Supplementary Fig. 1

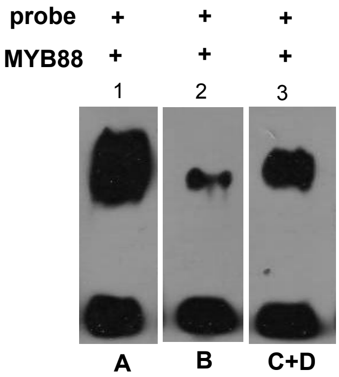

Figure S1. EMSA results show that MYB88 can bind *CDKA;1* promoter. Lane1, probes for binding Element A with His-MYB88 proteins; Lane 2, probes for Element B with His-MYB88 proteins; Lane 3, probes for Element C and D with His-MYB88 proteins.

Supplementary Fig. 2

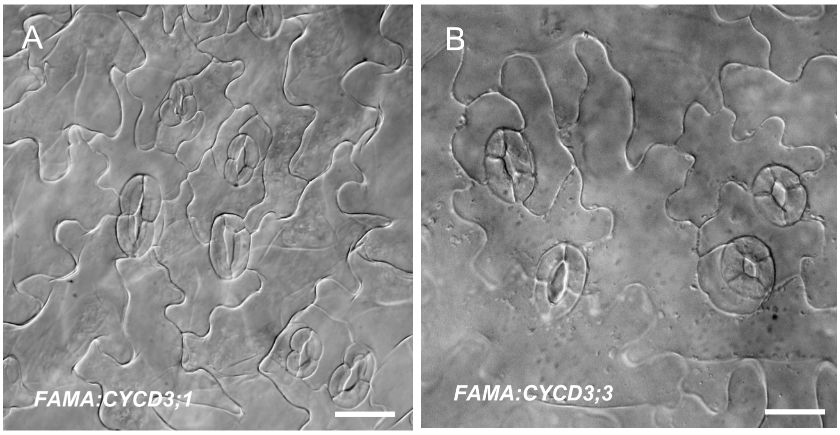

Figure S2. Expression of CYCD3;1 or CYCD3;3 promotes GC subdivisions. (A) *FAMA:CYCD3;1.* (B) *FAMA:CYCD3;3*. Bars: 20 μm.

Supplementary Fig. 3

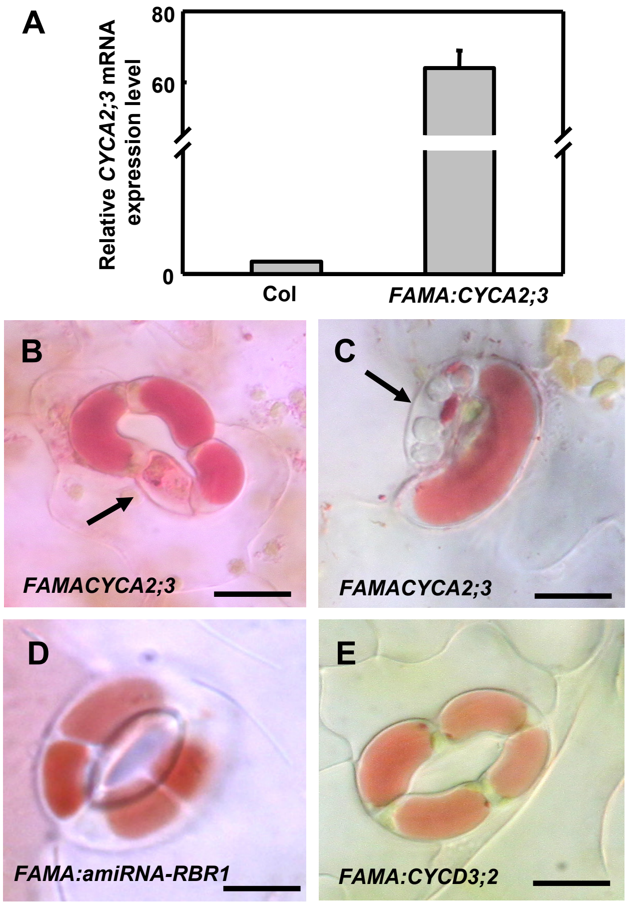

Figure S3. Overexpression of *CYCA2;3* induces GC subdivision and cell collapse. (A) RT-qPCR shows upregulation of *CYCA2;3* in *FAMA:CYCA2;3* seedlings. (B-E) Guard cell vitality is checked by neutral red staining. Neutral red dyes are taken by living cells and stored in vacuoles. Dead cells failed to take in dyes into vacuoles. Its cytoplasm debris was stained instead. Arrow points to the collapsed cells in *FAMA:CYCA2;3* (B, C). All GCs are vital in stomata from *FAMA:amiRNA-RBR1* (D) and *FAMA:CYCD3;2* (E). Bars: 10 µm.

Supplementary Fig. 4

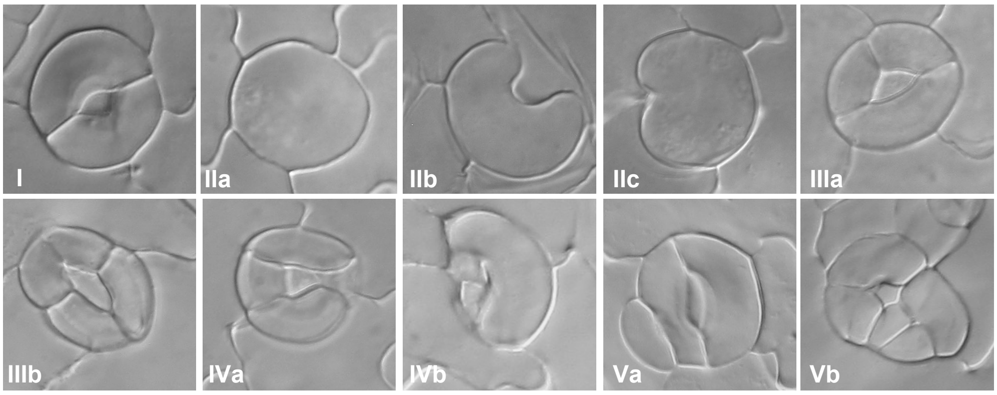

	Type I	Type II (a+b+c)	Type III (a+b)	Type Ⅳ (a+b)	Type Ⅴ (a+b)
Wild-type	100	0	0	0	0
FAMA:CYCA2;3	21	0	25	45	9
FAMA:CDKB1;1	100	0	0	0	0
FAMA:CYCA2;3 *FAMA:CDKB1;1*	23	0	23	42	12
35S:CDKB1.N146	87	13	0	0	0
FAMA:CYCA2;3 *35S:CDKB1.N146*	97.7	2.3	0	0	0
FAMA:CDKA;1	100	0	0	0	0
FAMA:CYCA2;3 *FAMA:CDKA;1*	17	0	32	49	10

Figure S4. Quantification of the synergistic effect of co-expression of *CYCA2;3* and *CDKB1;1* in promoting GC subdivisions. Type I, normal 2-GC stomata; Type II (a, b, c), SGC stomata; Type III (a, b) stomata with 3-4 radially orientated GCs; Type IV (a, b), stomata with collapsed cells; Type V (a, b) asymmetrically subdivided GCs.

Supplementary Fig. 5

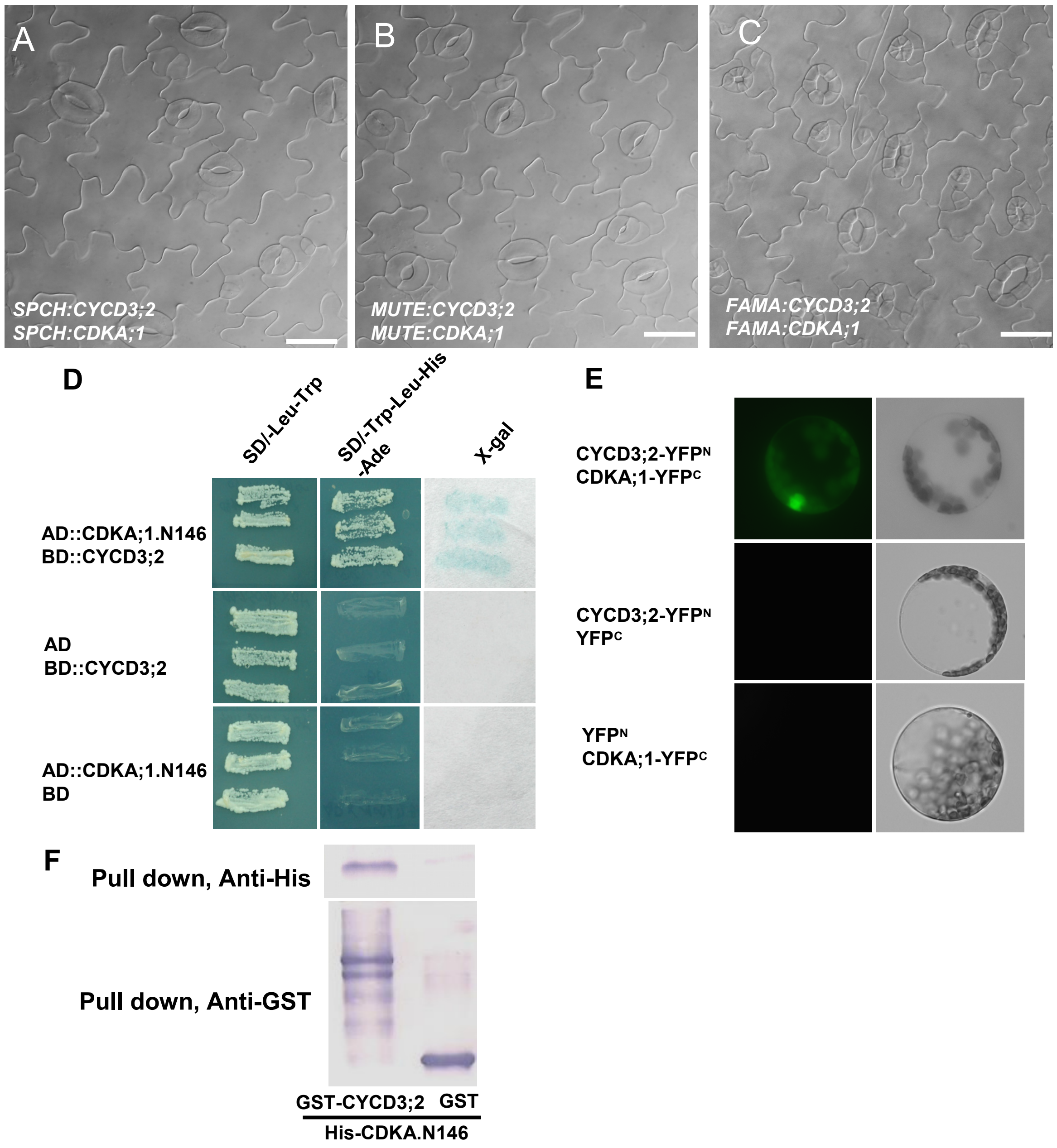

Figure S5. CDYD3;2 direct interacts with CDKA;1. (A, B) Co-expression *CDKA;1* and *CYCD3;2* under the control of either *SPCH* or *MUTE* promoter does not cause any visible stomatal defects. (C) Co-expression *CDKA;1* and *CYCD3;2* under the control of *FAMA* promoter leads aggregative extra GC subdivisions. (D) CDKA;1.N146 interacts with CYCD3;2 in a yeast two-hybrid analysis. The blue color indicates activation of reporter genes. AD, target construct vector; BD, bait construct vector. (E) The interaction between CDKA;1.N146 and CYCD3;2 is confirmed by a BiFC assay. YFP fluorescence was detected in the *Arabidopsis* protoplast when co-transformation with *CYCD3;2-YFP^N^* and *CDKA;1.N146-YFP^C^*, but not with *CYCD3;2-YFP^N^* and *YFP^C^*, or *YFP^N^* and *CDKA;1-YFP^C^*. (F) Pull-down assay indicates the direct interaction between CDKA;1.N146 and CYCD3;2.

Supplementary Fig. 6

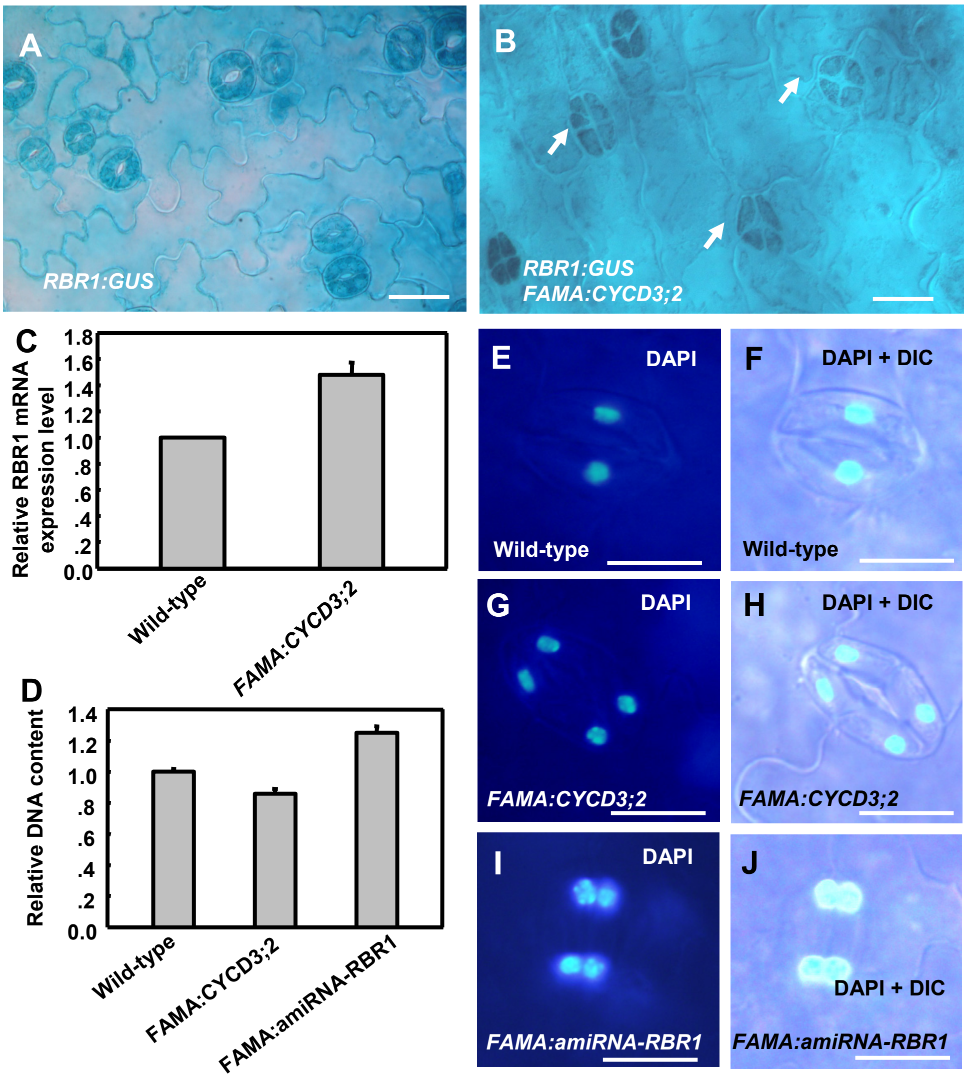

Figure S6. *RBR1* expression and GC subdivision*.* (A) *RBR1:GUS* expression in wild-type epidermal cells including stomatal lineage cells. (B) *RBR1:GUS* in *FAMA:CYCD3;2* background. Arrows indicate subdivided GCs. (C) RT-qPCR analysis show a slightly increased level of RBR1 in *FAMA:CYCD3;2.* (D) Quantification of DAPI-stained GCs in Wild-type, *FAMA:CYCD3;2,* and *FAMA:amiRNA-RBR1* transgenic plants. (E-J) DAPI staining reveals newly subdivided GCs containing a comparable DNA content with that of normal GCs.

Supplementary Fig. 7

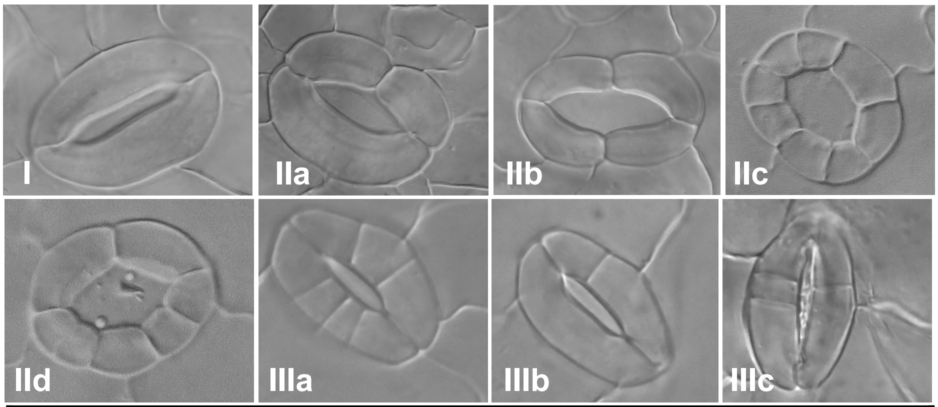

	Type I	Type II (a+b)	Type II (c+d)	Type III (a+b+c)	n
**Wild-type**	**100**	**0**	**0**	**0**	**568**
FAMA:CYCD3;2 FAMA:CDKA;1	**1**	**78**	**21**	**0**	**786**
FAMA:amiRNA-RBR1	**44.4**	**25.7**	**0**	**29.9**	**792**

Figure S7. Comparison of stomatal GC subdivision phenotypes in lines co-expression of *CYCD3;2* and *CDKA;1,* and *RBR1 RNAi* in promoting GC subdivisions in lines.
(A) Quantification of the synergistic effect of co-expression of *CYCD3;2* and *CDKA;1* in promoting GC subdivisions in *FAMA:amiRNA-RBR1* lines. Type I, normal 2-GC stomata. Type II a and b, 3- and 4-GC stomata. Type II c and d, stomata with more than 4 GCs. Type III a-c, extra divisions within the original GCs, notes the unchanged GC shape comparing with the “sausage chain”-like Type II stomata.

Table S1. Primer sequences used in this article.

Primer Name	Description	Sequence
CDKA;1-OF	*CDKA;1* cDNA	5'-GACTAGTATGGATCAGTACGAGAAAGTT-3'
CDKA;1-OR	*CDKA;1* cDNA	5'-CGAGCTCCTAAGGCATGCCTCCAAGATCC-3'
CYCD3;1-OF	*CYCD3;1* cDNA	5'-GACTAGTATGGCGATTCGGAAGGAGGAAG-3'
CYCD3;1-OR	*CYCD3;1* cDNA	5'-GGAGCTCTTATGGAGTGGCTACGATTGCC-3'
CYCD3;2-OF	*CYCD3;2* cDNA	5'-CTCTAGAATGGCTTTGGAGAAAGAGGAA-3'
CYCD3;2-OR	*CYCD3;2* cDNA	5'-GGAGCTCCTAGCGAGGACTACTAAGCACAT-3'
CYCD3;3-OF	*CYCD3;3* cDNA	5'-CTCTAGAATGGCTTTAGAAGAGGAGGAAG-3'
CYCD3;3-OR	*CYCD3;3* cDNA	5'-GGAGCTCTTAGCGAGGACTACTACTAAG-3'
CYCA2;3-OF	*CYCA2;3* cDNA	5'-GACTAGTATGGGGAAGGAAAATGCTGT G-3'
CYCA2;3-OR	*CYCA2;3* cDNA	5'-CGAGCTCTCAGAATAGCGTGTCAAGTAG-3'
CDKA;1.N146-yeast-1	Yeast-two hybrid	5'-GGAATTCATGGATCAGTACGAGAAAGTT-3'
CDKA;1.N146-yeast-2	Yeast-two hybrid	5'-GGTCGACCTAAGGCATGCCTCCAAGATC-3'
CYCD3;2-yeast-1	Yeast-two hybrid	5'-GGAATTCATGGCTTTGGAGAAAGAGGAAGA-3'
CYCD3;2-yeast-2	Yeast-two hybrid	5'- GGGATCCCTAGCGAGGACTACTAAGCAC-3'
CDKA;1.N146-BiFC-1	BiFC	5'-GACTAGTATGGATCAGTACGAGAAAGTT-3'
CDKA;1.N146-BiFC-2	BiFC	5'-GCCTCGAGAGGCATGCCTCCAAGATCCT-3'
CYCD3;2-BiFC-1	BiFC	5'-CTCTAGAATGGCTTTGGAGAAAGAGGAA-3'
CYCD3;2-BiFC-2	BiFC	5'-GCGGATCCGCGAGGACTACTAAGCACAT-3'
CDKA;1.N146- Pd-1	Pull-down	5'-GAATTCATGGATCAGTACGAGAAAGTT-3'
CDKA;1.N146- Pd-2	Pull-down	5'-GGTCGACCTAAGGCATGCCTCCAAGATC-3'
CYCD3;2- Pd-1	Pull-down	5'-GGAATTCATGGCTTTGGAGAAAGAGGAAGA-3'
CYCD3;2- Pd-2	Pull-down	5'-GGGATCCCTAGCGAGGACTACTAAGCAC-3'
SPCH-P1	SPCH promoter	5'-AATCCCCAACTAAAATATCCAACT-3'
SPCH-P2	SPCH promoter	5'-GCGGCCGCGTGATTAAGAGATATATCCT-3'
MUTE-P1	MUTE promoter	5'-TTGGTTTGGTTTTCGACCCTTG-3'
MUTE-P2	MUTE promoter	5'-CAAGATTCTCTTCTGGAGTTCC-3'
FAMA-P1	FAMA promoter	5'-CCTATATCCAAGTTTGAAGTACAGTT-3'
FAMA-P2	FAMA promoter	5'-CCTATATCCAAGTTTGAAGTACAGTT-3'
CYCD3;2-P1	CYCD3;2 promoter	5'-GCCTGCAGTGAAAGAATCTACGCATTACG-3'
CYCD3;2-P2	CYCD3;2 promoter	5'-CGTCTAGACTTCTTCTTCTTCACGGAAGA-3'
RBR-P1	RBR promoter	5'-GCAAGCTTGAGCCACTAGGAATTTGCCTCG-3'
RBR-P2	RBR promoter	5'-GCACTAGTAGTCTCCAACGCAGCTGAAAAC-3'
RBR-real-time-1	Real time	5'-AGTGAAGAGGCTTAGTGTGAG-3'
RBR-real-time-2	Real time	5'-TCCACAATATTTAGCTTCAGAGCC-3'
CDKA;1-real-time-1	Real time	5'-GCCAAAAGCCCTTATTTCCT-3'
CDKA;1-real-time-2	Real time	5'-GTTACCCCACGCCATGTATC-3'
CYCD3;2real-time-1	Real time	5'-AATGGCACCAGCAATTAGACTT-3'
CYCD3;2real-time-2	Real time	5'-TTCAACTTCATCACATGGCTTC-3'
CYCA2;3real-time-1	Real time	5'-ACACTCCCACTCCAAAAACG-3'
CYCA2;3real-time-2	Real time	5'-AGCAACAACGGAAGGAAGAA-3'

FLP protein-1	Protein expression	5'-cggaattcATGGAAGATACGAAGAAGAAA-3'
FLP protein-2	Protein expression	5'-ccctcgagTTACAAGCTATGGAGAAGGAC-3'
MYB88 protein-1	Protein expression	5'-CGGAATTCATGGAAGAGACAACTAAGCAG-3'
MYB88-protein-2	Protein expression	5'-ccctcgagTTACAAGCTATCGAGAAGGAC-3'
CDKA;1-p-a-1	Y1H	5'-GCGAATTCCGGTATAATAAATAAAATTGTAC-3'
CDKA;1-p-a-2	Y1H	5'-GCCTCGAGGCGACGATCGGATGTAGTGTC-3'
CDKA;1-p-b-1	Y1H	5'-GCGAATTCCCTCTCTTCAAGCTTTTCACAGA-3'
CDKA;1-p-b-2	Y1H	5'-GCCTCGAGAGGACTAGGGAAGGGGATTGAC-3'
pCDKA-A_F	EMSA	5'-TAATAAATAAAATTGTACCAAATTAATGCGGTTAG GTAAATTCAATTTATTGGAAATTA-3'
pCDKA-A_R	EMSA	5'-TAATTTCCAATAAATTGAATTTACCTAACCGCATT AATTTGGTACAATTTTATTTATTA-3'
pCDKA-B_F	EMSA	5'-CTTGACACAAAAAAAAAAAAAGAAGAAAGAAAA AACCCTTACTCTCAATTAGTCAATCC-3'
pCDKA-B_R	EMSA	5'-GGATTGACTAATTGAGAGTAAGGGTTTTTTCTTT CTTCTTTTTTTTTTTTTGTGTCAAG-3'
pCDKA-C+D_F	EMSA	5'-TTACTCTCAATTAGTCAATCCCCTTCCCTAGTCC TCCTCTCTTCAAGCTTTTCACAGAA-3'
pCDKA-C+D-R	EMSA	5'-TTCTGTGAAAAGCTTGAAGAGAGGAGGACTAG GGAAGGGGATTGACTAATTGAGAGTAA-3'
pCDKA-Cm_F	EMSA	5'-TTACTCTCAATTAGTCAAAAAAATTCCCTAGTCC TCCTCTCTTCAAGCTTTTCACAGAA-3'
pCDKA-Cm_R	EMSA	5'-TTCTGTGAAAAGCTTGAAGAGAGGAGGACTAG GGAATTTTTTTGACTAATTGAGAGTAA-3'
pCDKA-Dm_F	EMSA	5'-TTACTCTCAATTAGTCAATCCCCAAAAATAGTCC TCCTCTCTTCAAGCTTTTCACAGAA-3'
pCDKA-Dm_R	EMSA	5'-TTCTGTGAAAAGCTTGAAGAGAGGAGGACTATT TTTGGGGATTGACTAATTGAGAGTAA-3'
Lba1	Genotyping	5'-TGGTTCACGTAGTGGGCCATCG-3'
SALK_106809-LP	Genotyping	5'-TCAAACAAGTTTGGTTTTGGC-3'
SALK_106809-RP	Genotyping	5'-TTCCTTGTTCATATGTTCCCG -3'
